# Radiomic features of PET/CT imaging of large B cell lymphoma lesions predicts CAR T cell therapy efficacy

**DOI:** 10.3389/fonc.2024.1485039

**Published:** 2024-11-25

**Authors:** Yoganand Balagurunathan, Zhouping Wei, Jin Qi, Zachary Thompson, Erin Dean, Hong Lu, Saran Vardhanabhuti, Salvatore Corallo, Jung W. Choi, Jenny J. Kim, Mike Mattie, Michael Jain, Frederick L. Locke

**Affiliations:** ^1^ Department of Machine Learning, H. Lee. Moffitt Cancer Center, Tampa, FL, United States; ^2^ Department of Cancer Physiology, H. Lee. Moffitt Cancer Center, Tampa, FL, United States; ^3^ Department of Biostatistics and Bioinformatics, H. Lee. Moffitt Cancer Center, Tampa, FL, United States; ^4^ Blood and Marrow Transplant, H. Lee. Moffitt Cancer Center, Tampa, FL, United States; ^5^ Medicine in the Division of Hematology and Oncology, University of Florida, Gainesville, FL, United States; ^6^ Department of Breast Imaging, Tianjin Medical University Cancer Institute & Hospital, Tianjin, China; ^7^ Clinical Research, Kite Pharma, a Gilead Company, Santa Monica, CA, United States; ^8^ Department of Diagnostic Imaging & Interventional Radiology, H. Lee. Moffitt Cancer Center, Tampa, FL, United States

**Keywords:** imaging biomarkers in lymphoma, MTV (metabolic tumor volume), radiomics in immunotherapy, PET/CT scan, biomarkers in CAR T cell therapy

## Abstract

**Background:**

Relapsed and refractory Diffuse large B cell lymphoma (DLBCL) can be successfully treated with axicabtagene ciloleucel (axi-cel), a CD19-directed autologous chimeric antigen receptor T cell (CAR-T) therapy. Diagnostic image-based features could help identify the patients who would clinically respond to this advanced immunotherapy.

**Purpose:**

The aim of this study was to establish a radiomic image feature-based signature derived from positron emission tomography/computed tomography (PET/CT), including metabolic tumor burden, which can predict a durable response to CAR-T therapy in refractory/relapsed DLBCL.

**Methods:**

We conducted a retrospective review of 155 patients with relapsed/refractory DLBCL treated with axi-cel CAR-T therapy. The patients’ disease involvement was evaluated based on nodal or extranodal sites. A sub-cohort of these patients with at least one nodal lesion (n=124) was assessed, while an overlapping sub-cohort (n=94) had at least one extranodal lesion. The lesion regions were characterized using 306 quantitative imaging metrics for PET images and CT images independently. Principal component (PC) analysis was performed to reduce the dimensionality in feature-based functional categories: size (n=38), shape (n=9), and texture (n=259). The selected features were used to build prediction models for survival at 1 year and tested for prognosis to overall/progression-free survival (OS/PFS) using a Kaplan-Meier (KM) plot.

**Results:**

The Shape-based PC features of the largest extranodal lesion on PET were predictive of 1-year survival (AUC 0.68 [0.43,0.94]) and prognostic of OS/PFS (p<0.018). Metabolic tumor volume (MTV) was an independent predictor with an area under the curve (AUC) of 0.74 [0.58, 0.87]. Combining these features improved the predictor performance (AUC of 0.78 [0.7, 0.87]). Additionally, the Shape-based PC features were unrelated to total MTV (Spearman’s ρ of 0.359, p≤ 0.001).

**Conclusion:**

Our study found that shape-based radiomic features on PET imaging were predictive of treatment outcome (1-year survival) and prognostic of overall survival. We also found non-size-based radiomic predictors that had comparable performance to MTV and provided complementary information to improve the predictability of treatment outcomes.

## Introduction

Non-Hodgkin’s lymphoma (NHL) accounts for approximately 4% of all cancers, with over 20,250 deaths in 2021 in the United States (1–3). Diffuse large B cell lymphoma (DLBCL) is the most common form of aggressive NHL, accounting for 30% to 50% of cases (4, 5). DLBCL is genetically and biologically heterogeneous, with variable therapy responses (6–8). Axicabtagene ciloleucel (axi-cel), a CD19-targeted chimeric antigen receptor (CAR)-T cell therapy, has revolutionized the treatment of the disease, demonstrating superior levels of durable response in relapsed/refractory (R/R) DLBCL (9–11). As only a proportion of patients respond to this relatively new therapy, developing prognostic biomarkers is a critical unmet clinical need.

Total metabolic tumor volume (MTV) measured on ^18^F fluorodeoxyglucose (^18^F-FDG) positron emission tomography/computed tomography (PET/CT) has been associated with treatment outcomes, and the assessment involves a semi-automated process with clinical oversight to avoid false detection (12, 13). Recent advancements in imaging methodologies have made it possible to utilize medical images (PET/CT) to develop quantitative biomarkers using radiomic metrics (14–16), further extended by artificial intelligence (AI) methods (17, 18). Recently, radiomic metrics on PET imaging have been shown to be prognostic for complete response (19) and predict clinical benefit after CAR-T treatment (20).

In this study, we used advanced quantitative imaging metrics (radiomics) on PET/CT images of DLBCL tumors to identify imaging observed metrics that may predict treatment response (1-year survival), specifically to CAR-T therapy. We compared our findings with whole-body MTV. Our study overview is illustrated in **Supplementary Figure S1**.

## Methods

### Patient cohort

We retrospectively obtained patient records after approval from our Institutional Review Board at the University of South Florida/Moffitt Cancer Center (MCC). Patients with R/R DLBCL between May 2015 to June 2019 at MCC (n=100), at consortium sites between November 2015 to September 2016 (n = 55), and who had received axi-cel treatment as a third or later line of therapy were enrolled. In the study, 55 of 155 patients were part of the cohort from a previously reported consortium trial (10). This previous article showed the effectiveness of CAR T cell immunotherapy (axi-cel) after the failure of conventional therapy, with a reported objective response rate of 82% and a complete response rate of 54% with a median follow-up of 15.4 months. Clinical parameters for the consortium patients (n=55) were blinded from the study authors. We obtained imaging scans (18F-FDG PET/CT) and clinical data from these patients. Patients without baseline imaging (^18^F FDG-PET/CT) prior to CAR T cell therapy were excluded. Patients may have received bridging therapy as a standard of care (SOC), defined as any lymphoma-specific therapy given after apheresis but before the start of fludarabine and cyclophosphamide chemotherapy for lymphodepletion before the CAR-T infusion (see **Table 1A**).

**Table 1:** Patient data used for radiomic analysis: A) clinical and demographic characteristics, B) lesions categorized by anatomical location, and C) PET/CT scanner types.

TABLE 1A. Cohort clinical characteristics.			
Characteristic	All patients* (N=155)	Lesion site used for radiomics	
		Extranodal (n=94)	Lymphatic (n=124)
**Age** (mean, median, std.dev)	60.1 (63, 12.2)	59.4 (63.5, 12.8)	61 (63, 10.9)
**Sex** (male/female/unavailable)	61/39/55	36/22	53/29
**LDH** (mean, median, std.dev)	400.5 (266, 348.25)	448.3 (275.5, 406.79)	408.6 (267.5, 353.4)
ECOG			
0-1	83	48	66
2-3	17	10	16
One-year progression or death: No Yes		41 (43.6%) 53 (56.4%)	61(49.2%) 63 (50.8%)
Stage			
I/II III/IV Unavailable	22 78 55	10 48	14 68
Bridge therapy			
Yes No Unavailable	Yes: 50 No: 50 55	Yes: 28 No: 30	Yes: 41 No: 41
Axi-cel administration			
Trial (cancer center) Zuma-1	100 55	58 36	82 42

TABLE 1B. Lesions categorized by anatomical location.				
	Lymphatic (n = 124)		Extranodal (n = 94)	
#	Anatomical Location	# Patients (%)	Anatomical Location	# Patients (%)
1	Abdomen	58 (46.77%)	Abdomen	3 (3.19%)
2	Lungs (Chest)	8 (6.45%)	Lungs (Chest)	35 (37.23%)
3	Pelvis	22 (17.74%)	Pelvis	8 (8.51%)
4	Musculoskeletal system (bone or muscle)	0 (0%)	Musculoskeletal system (bone or muscle)	20 (21.28%)
5	Mediastinum (Chest)	11 (8.87%)	Mediastinum (Chest)	0 (0%)
6	Neck	10 (8.06%)	Neck	0 (0%)
7	Liver	0 (0%)	Liver	7 (7.45%)
8	Axilla	5 (4.03%)	Axilla	0 (0%)
9	Spleen	1 (0.81%)	Spleen	4 (4.26%)
10	Leg	1 (0.81%)	Leg	4 (4.26%)
11	Cutaneous (Skin)	0 (0%)	Cutaneous (Skin)	4 (4.26%)
12	Breast	2 (1.61%)	Breast	1 (1.06%)
13	Colon	2 (1.61%)	Colon	1 (1.06%)
14	Neck	2 (1.61%)	Neck	1 (1.06%)
15	Arm	0 (0%)	Arm	2 (2.13%)
16	Stomach	1 (0.81%)	Stomach	1 (1.06%)
17	Hilar	1 (0.81%)	Hilar	0 (0%)
18	Mouth	0 (0%)	Mouth	1 (1.06%)
19	Pancreas	0 (0%)	Pancreas	1 (1.06%)
*Sub-total (Lymphatic)*		*124*	*Sub-total (Extranodal)*	*94*
*Some clinical variables were unavailable for consortium patients (n=55, see Methods section).				

TABLE 1C. PET/CT scanner details (n=155).			
	Manufacturer	Model (Count)	Count
1	Siemens	Biograph 16 (3)	21
		Biograph 64 (8)	
		Biography 40 (10)	
2	Phillips	Gemini (7)	11
		Ingenuity (2)	
		Brilliance64 (2)	
3	GE Medical System	DiscoverySTE (54)	123
		DiscoveryMR DR (44)	
		Discovery600 (5)	
		Discovery690 (3)	
		Discovery710 (3)	
		DiscoveryRX (2)	
		DiscoveryST (4)	
		DiscoverySTE (8)	
Sub-total			155

### PET/CT imaging

PET/CT scans are the primary modality used in disease staging of lymphoma disease as recommended by Lugano’s disease classification, adapted from the oncology guidelines (21, 22). The CT scan modality provides the anatomical details of the lesion’s morphology while the PET scan allows the assessment of contrast uptake that enables us to distinguish an abnormal lesion from a normal tissue (23). Patients undergo a standard-of-care whole-body CT with PET imaging to evaluate the disease condition prior to CAR-T treatment (baseline scan). The patient cohort was scanned on mixed clinical PET/CT scanners (see **Table 1C**). Images constructed with attenuated corrected (AC) scans were used for the study as it has been shown that AC PET imaging allows appropriate intensity scaling (24). The voxel values were converted to standardized uptake values (SUVs) prior to feature extraction, followed by statistical analysis.

### Clinical features

Lactate dehydrogenase (LDH) levels are known to be associated with tissue damage due to disease, infection, or injury. They have been shown to be significantly elevated in both indolent and aggressive non-Hodgkin’s lymphoma (25). We used serum-based (baseline) estimates in our prognostic model to complement the metabolic tumor burden and radiomic metrics.

### Metabolic tumor burden computation

We used the patient’s baseline ^18^F-FDG PET/CT scans obtained prior to axi-cel treatment to evaluate MTV using custom tools implemented on MIM Software, our research PACS (version 6.8.4, MIM Software, Cleveland, OH). The semi-automated workflow requires the user to identify a normal hepatic (liver) reference using a single selection click. Using the selected location, a 2-cm sphere region is automatically drawn to estimate the statistics of the reference region. Following the Positron Emission Tomography Response Criteria (PERCIST) recommendations (26), regions over two standard deviations from reference (normal hepatic or liver regions) were automatically identified, delineated, and stored. These identified regions were individually evaluated (manual process) by a clinical expert (radiologists J.Q. and J.W.C., and oncologist, E.A.D.) to discriminate and remove physiologically active regions (brain/bladder/etc.) and regions with perceived non-oncologic inflammation or infection. After the manual evaluation, voxels over 41% of SUVmax at the lesion level were converged (as PERCIST recommended). These regions were then summated across the body to obtain MTV at the patient level, reported in milliliters (mL), as previously assessed and presented (12).

### Lesion review

Lesions that were validated and part of the MTV computation were re-reviewed by our research/clinical radiologists (J.Q/J.W.C) to capture additional details about them: i) anatomical location, ii) individual metabolic volumes of the lesions at the patient level, and iii) association of the lesions with the lymphatic system (lymphatic vs. extranodal). The lesions were broadly categorized by the anatomical location (20 sites in total), for the patient’s largest lesions. We selected the largest lymph node (in lymphatic-related lesions) and the largest nodule (in extranodal-related lesions) in each patient. Thus, we assembled a sub-cohort with 124 patients with their largest lesion related to the nodal disease (lymph node) and another with 94 patients with their largest lesion related to extranodal disease. The organ site information about the largest lesion in each patient which was utilized for further radiomic analysis is provided in **Table 1B** with more details described in the Results section. It is possible that patients can be part of both or either of the cohorts (lymphatic or extranodal).

### Radiomics

CT and FDG-PET imaging were re-sampled to the same resolution of 1x1x1 mm^3^ using bilinear interpolation and then standardized to SUV units using the activity concentration calibrated to the dosage of ^18^F-FDG injected volume and patient body weight after decay correction, using custom tools developed in Matlab, Mathworks Inc ^®^. The regions that converged to be metabolically active (using the same procedure followed for MTV) were used as the lesion boundary in the PET images and mapped to CT images. These boundaries were used to extract 306 radiomic features in each modality (CT and PET), totaling 612 features obtained to characterize each lesion across modalities. Based on clinical assessment, the largest metabolic lesion (based on PET volume) associated with lymphatic and extranodal regions were selected. These nodules across the patients were sorted to form sub-cohorts for analysis. We categorized imaging radiomic features into three broad functional categories: Size (n=38), Shape (n =9), and Texture (n=259), with details on the features differed to **Supplementary Tables S1**–**S3**. The radiomic feature classification is based on the functional nature of the descriptors. It follows conventional formulations and definitions in adherence to the recommendations of the Image Biomarker Standardization Initiative (IBSI) consensus criteria (27).

### Feature dependencies

The dependency between the radiomic features and whole-body MTV was evaluated by computing correlation coefficient metrics. We repeated the comparison across the modalities (PET/CT), sub-cohorts (Lymphatic and Extra-nodal), and for the principal components (PCs) calculated at the feature category level (Size, Shape, and Texture). The highly correlated features were ordered (descending) across the modalities (PET/CT) and sub-cohorts (Lymphatic, Extra-nodal). We found that the Texture-based feature, PC1 (first principal component), in the Lymphatic group (CT features) had the lowest correlation (ρ =0.35), followed by the Texture PC1 feature in the Extranodal group (CT features, ρ =0.38). The Texture PC7 (seventh principal component) feature in the Lymphatic group (PET features) had a moderate negative correlation (ρ = -0.33), and the Shape feature PC1 (Shape PC1) in the Extranodal group (PET features) had a moderate correlation (ρ = 0.36) (see **Supplementary Table S4**). Shape-based PCs tended toward and away from the Size-based PCs and overall Metabolic Tumor Volume (MTV), respectively (see **Supplementary Figure S2**).

### Statistical analysis

A logistic regression model was used to test the predictive ability of the imaging features. The features with the lowest change (coefficient of variance ≤3%) across the patient samples were removed. The PCs in each of the feature categories were computed using the first three components (1 to 3) to build a logistic regression model. The relationship between the radiomic feature-based principal components and MTV in each category across the imaging modality was assessed using Spearman’s correlation coefficients (ρ) (see **Supplementary Table S4**). A logistic regression model was constructed using MTV and radiomic and clinical features to predict 1-year survival after treatment. The PCs were computed using radiomic features in each functional category (Size, Shape, Texture) and models were assessed independently using these feature metrics. The model performance was measured by sensitivity, specificity, and area under receiver operator characteristics (AUC), estimated by 5-fold cross-validation. In the final analysis, we used the logistic regression model based on the PCs (PC1-3) in the non-size-based functional categories (shape, texture) to divide the cohort into risk groups. We assessed the risk of disease progression measured by overall survival using the Cox regression model. We measured the ability of features to predict overall survival (OS) and progression-free survival (PFS) using Kaplan-Meier (KM) plots, and the log-rank test was used to assess the significance. A p-value of less than 0.05 was considered statistically significant in our analysis.

## Results

**Figure 1:**
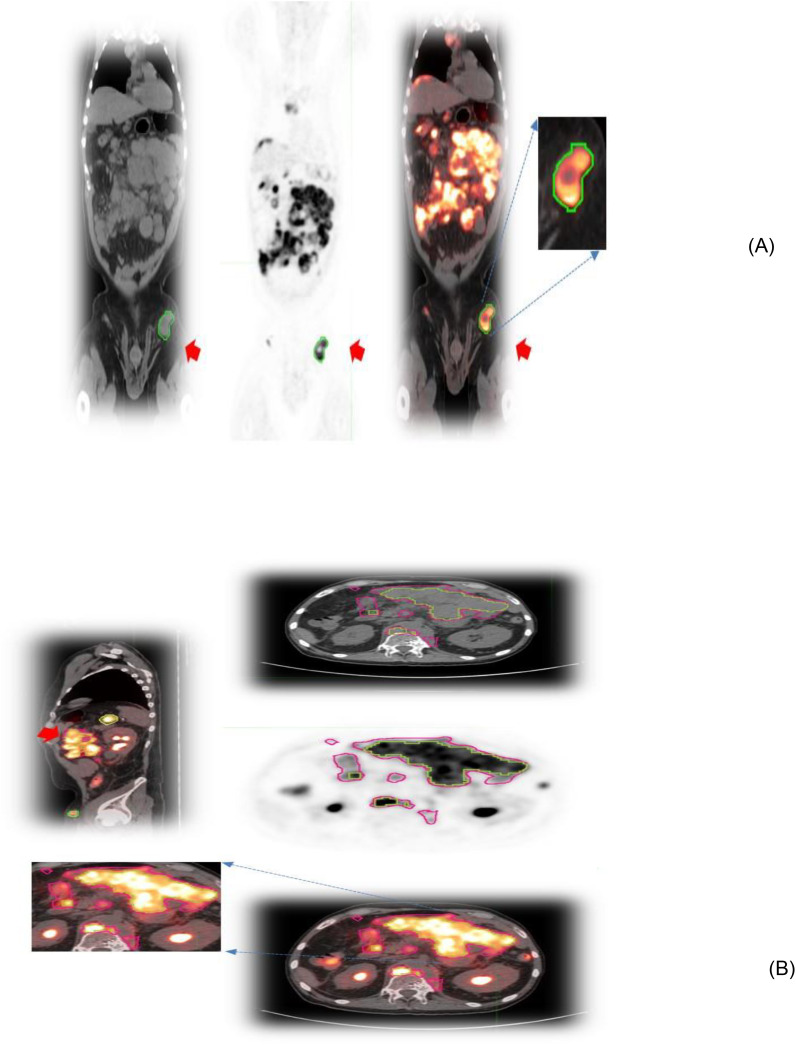
Patient scans showing representative slices of lesions in different image modalities (CT/PET and fused) with an arrow. **(A)** Lesions associated with the lymphatic system in the pelvis and **(B)** extranodal lesions in the abdomen.

In this study, we created a cohort of 155 patients who were treated with axi-cel therapy. Baseline radiological scans (PET/CT) scans were obtained and there were 1,639 lesions assessed across the patient cohort that our clinical radiologist manually verified. Of these, 1,058 lesions were detected in 100 patients (MCC) and 581 lesions were detected in 55 patients from ZUMA-1 (consortium). The normal (benign) hepatic (liver) reference region was excluded from the counts. We formed sub-cohorts with patients with their largest lesion related to either lymphatic (n=124) or extranodal (n=94) disease. **Figure 1** shows lymphatic (i.e., nodal) and extranodal lesions. The most frequently involved nodal sites were the abdomen (46.77%, n=58), pelvis (17.74%, n=22), mediastinum (8.87%, n=11), neck (8.06%, n=10), and lungs (5.65%, n=7). The most frequently involved extranodal sites were the lungs (36.17%, n=34), musculoskeletal system (21.28%, n=20), pelvis (8.51%, n=8), liver (7.45%, n=7), and spleen (4.26%, n=4), (**Table 1B**). The fifth most common extranodal site in our cohort was evenly shared between the spleen, legs, and skin, with approximately 4.26% of patients (n=4). In this study, radiomic features were categorized into major functional characteristics, i.e., Size, Shape, and Texture (n=259 features) (**Supplementary Table S1**), and feature metrics were assessed independently in CT and PET scans. We reviewed the relationship of principal components across three categories (Size, Shape, Texture) with MTV and individual lesion volume, displayed as a scatter plot of the principal components, PC1 and PC2, for extranodal CT features and extranodal PET features, respectively (**Supplementary Figure S2**).

The dependency between the radiomic features and whole-body MTV was evaluated by correlating these metrics at the category level, and we found that the Texture feature PC1 (first principal component) in the Lymphatic group (CT features) had the lowest correlation (ρ =0.35), followed by the Texture PC1 feature in the Extranodal group (CT features, ρ =0.38). The details are given in **Supplementary Table S4**, **Supplementary Figure S2**.

**Figure 2:**
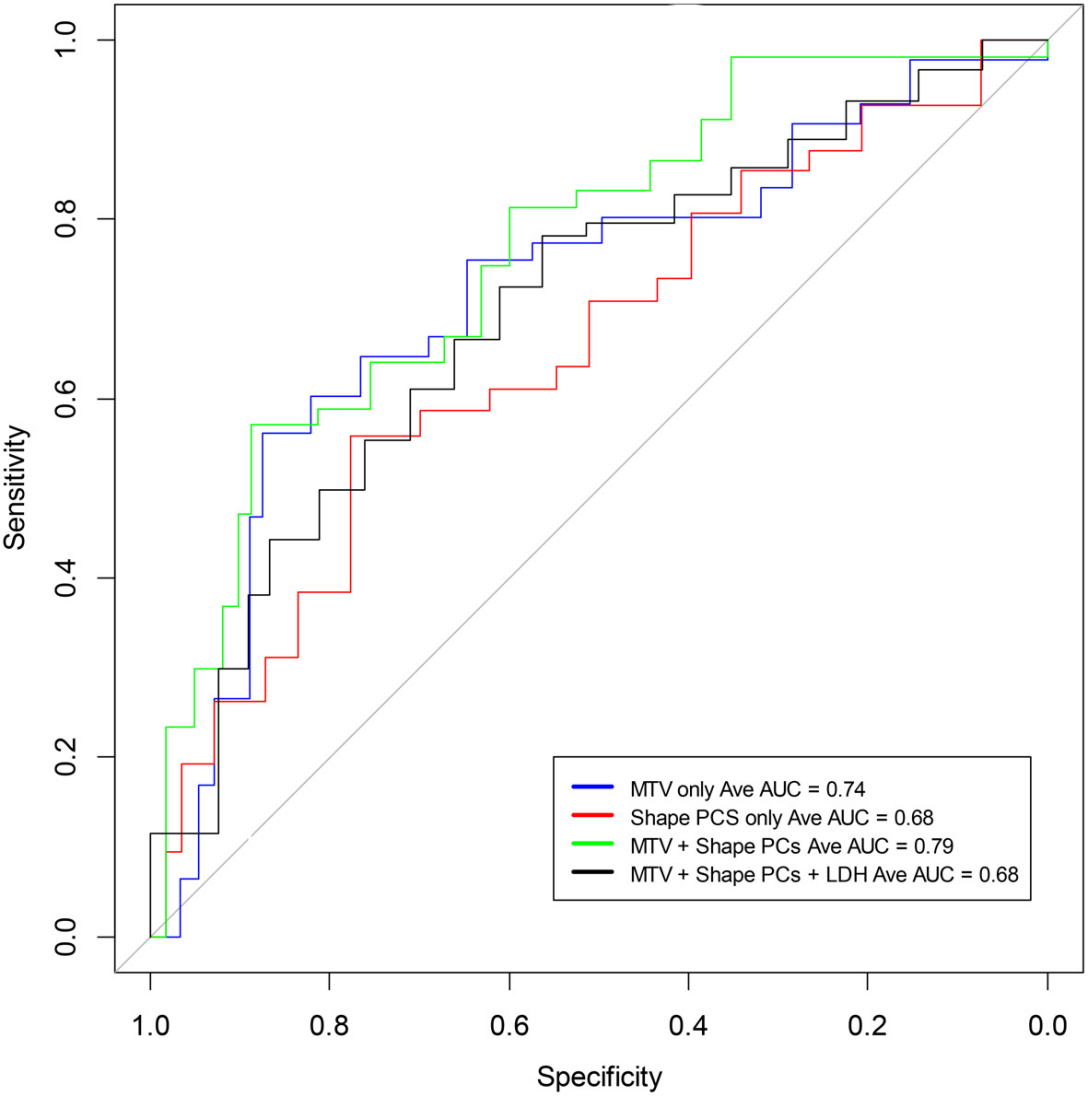
Receiver operating characteristic curves to predict 1-year overall survival using a logistic model based on shape features of the largest lesion in the extranodal regions of PET (SUV) images (see **Table 2**). The curves include MTV (average AUC 0.74), the Shape-based radiomic features (Principal components 1 to 3) (average AUC 0.68), and Shape-based radiomic features (Principal components 1 to 3) with MTV (average AUC 0.79).

**Figure 3:**
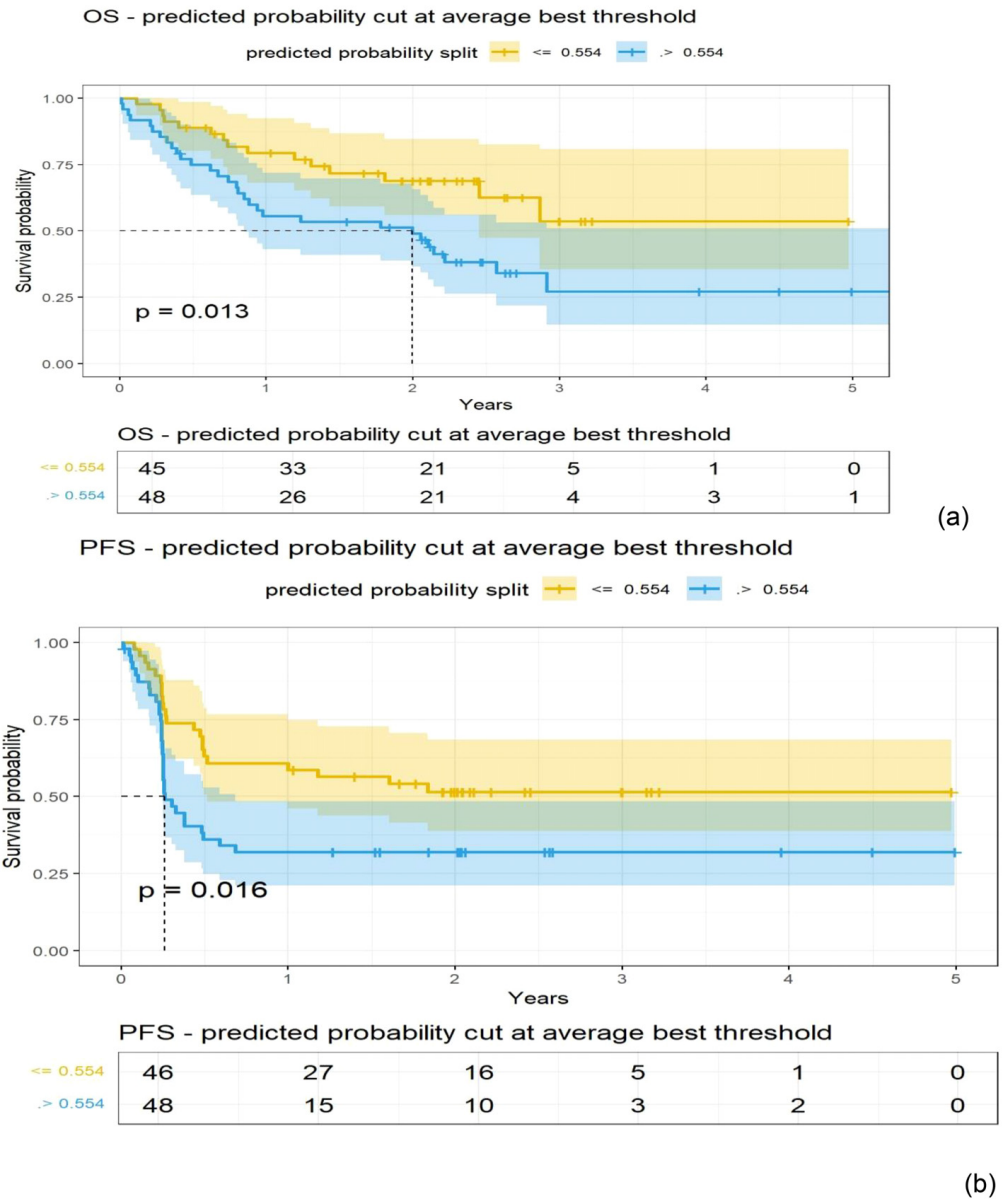
Kaplan-Meier (KM) plots obtained using patients grouped with a cut-off point obtained from a logistic model for the Shape-based radiomic features (Principal components 1 to 3) extracted from the extranodal regions of PET (SUV) images (details in **Table 2**); **(A)** Overall survival, **(B)** Progression-free survival.

**Figure 4:**
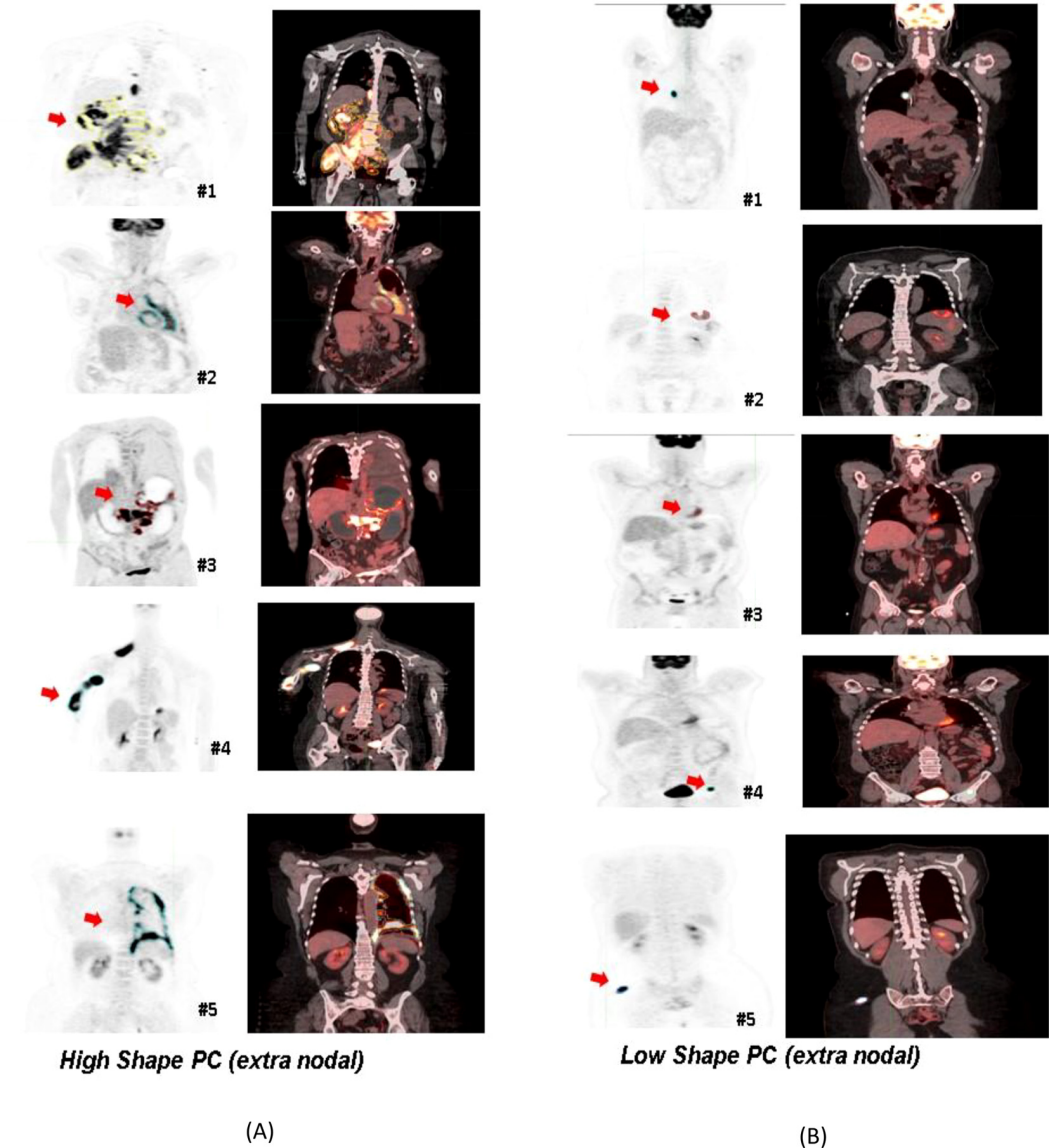
Patients selected based on shape-based predictors (principal component-based) using the PET radiomic features of their largest extranodal lesion. Representative PET/Fused (PET-CT) image slices were selected: **(A)** The panel on left side indicated high Shape PC metric, and **(B)** the panel on the right corresponds to patients with a smaller value for the Shape PC metric for their largest extranodal lesion.

A logistic regression model was built using total body MTV and radiomic feature-based PCs to predict survival (1 year) after treatment. We found that total body MTV remains predictive with an average AUC of 0.71 to 0.76 across the different sub-cohorts (see **Tables 2A–D**). We built a logistic model using the Shape-based PCs, which were computed on CT (Lymphatic group) and MTV, either independently or in combination. We found that MTV shows better predictive value than the Shape-based PCs, with an average AUC of 0.72 and 0.53, respectively, with the combination not showing an improvement (average AUC of 0.67) (see **Table 2A**). In the PET SUV (Lymphatic group) image cohort, using Shape-based PC features had an average AUC of 0.61, and the MTV-based predictor yielded an average AUC of 0.71. The combination of these metrics marginally improved the predictive value (average AUC to 0.72), while adding LDH did not improve the predictive ability (AUC of 0.62) (see **Table 2B**). Using CT image-based features (Extranodal group) did not show predictive value improvement with the addition of MTV (Shape-based PCs avg. AUC was 0.57 [0.31,0.83], MTV was 0.76 [0.62,0.89], while the combination was 0.46 [0.33,0.60], and with LDH it was 0.697 [0.51, 0.89]) (see **Table 2C**). Using PET SUV image metrics (Extranodal group) had an average AUC of 0.68[0.53,0.82], while MTV had an average AUC of 0.74[0.58, 0.89], and the combination showed an improved average AUC of 0.78 [0.7, 0.87]; however, the addition of LDH did not improve the predictive value (avg. AUC of 0.68 [0.42,0.94]) (see **Table 2D**, **Figure 2**). We further divided the cohort into lower tumor burden (lower median MTV) and higher tumor burden (greater than median MTV). We repeated the predictive analysis to evaluate the role of radiomic metrics. We found that the Shape-based PCs showed a comparable predictive value of AUC 0.716 to MTV (AUC of 0.75), (see **Table 2A**). The 1-year OS predictive ability did not improve in combination (Shape-based PCs and MTV). Furthermore, in patients with higher tumor burden, MTV and the Shape-based PC did not seem to be good predictors of 1-year OS (see **Table 2B**). We found that the Texture-based PCs showed a similar trend to the Shape-based PCs. The Texture-based PCs computed in CT (Lymphatic group), PET SUV (Lymphatic group), and CT (Extranodal group) did not improve on MTV-based predictors in the respective cohorts (see **Supplementary Tables S8A–C**). When using the Texture-based PCs computed on PET SUV (Extranodal group), the metrics had a moderate predictive ability, with an average AUC of 0.59 compared to MTV-based predictors (AUC of 0.74), while the combination showed a moderate improvement (average AUC of 0.66) (see **Supplementary Figure S3**, **Supplementary Table S8D**). We tested for prognosis using KM plots by grouping patients based on the logistic model and found that the Shape-based PC and Texture-based PC models were significant (P<0.001) for overall and progression-free survival (see **Figure 3**; **Supplementary Figure S4**). Using Shape features on PET images (Non-lymphatic group) had an 18% increased risk of disease progression compared to 15% using MTV, with a CI of 1.018, 1.371 and 1.038, 1.281, respectively (see **Supplementary Table S6**). The patients in our study had a median follow-up of 1 year after CAR-T treatment. **Figure 4** shows representative patient scans selected based on Shape-based PC predictors (high and low) for extranodal PET features.

**Table 2:** Logistic regression model to predict 1-year response to CAR-T therapy using whole-body metabolic tumor volume and radiomic PCs (Shape PCs) in patient image scans that are associated with A) Lymphatic-CT, B) Lymphatic-PET, C) Extranodal-CT, and D) Extranodal-PET observed across the cohort. The estimates were obtained using 5-fold cross-validation.

A1. Logistic regression using Shape-based PCs on CT Images (Lymphatic group)					
	Variable	Sensitivity/Specificity	E[AUC]	Prognosis	
				OS (p-val)	OS (p-val)
1	MTV	0.737 (0.608, 0.866)/0.737 (0.531, 0.943)	0.719 (0.572, 0.866)	<0.0001	0.0006
2	Shape-based PCs (1 to 3)	0.656 (0.299, 1)/0.589 (0.305, 0.873)	0.526 (0.400, 0.652)	0.8200	0.6600
3	MTV with Shape-based PCs (1 to 3)	0.769 (0.455, 1)/0.663 (0.375, 0.951)	0.677 (0.527, 0.827)	0.0019	0.0120
4	MTV with Shape-based PCs (1 to 3) and LDH	0.726 (0.576, 0.876)/0.679 (0.454, 0.904)	0.662 (0.607, 0.717)	0.0005	0.011

B1. Logistic regression using Shape-based PCs on PET Images (Lymphatic group)					
	Variable	Sensitivity/Specificity	E[AUC]	Prognosis	
				OS (p-val)	OS (p-val)
1	MTV (whole-body)	0.676 (0.446, 0.906)/0.773 (0.598, 0.948)	0.711 (0.604, 0.818)	<0.0001	0.0035
2	Shape-based PCs (PC 1 to 3)	0.72 (0.535, 0.905)/0.693 (0.512, 0.874)	0.639 (0.576, 0.702)	0.27	0.51
3	MTV, Shape-based PCs (1 to 3)	0.761 (0.707, 0.815)/0.752 (0.656, 0.848)	0.718 (0.68, 0.756)	0.0004	0.0005
4	MTV, Shape-based PCs (1 to 3) and LDH	0.615 (0.423, 0.807)/0.786 (0.557, 1)	0.662 (0.565, 0.759)	<0.0001	<0.0001

C1. Logistic regression using Shape-based PCs on CT Images (Extranodal group)					
	Variable	Sensitivity/Specificity	E[AUC]	Prognosis	
				OS (p-val)	OS (p-val)
1	MTV (whole-body)	0.709 (0.406, 1)/0.88 (0.715, 1)	0.758 (0.622, 0.894)	<0.0001	0.0018
2	Shape-based PCs (PC 1 to 3)	0.641 (0.477, 0.805)/0.698 (0.399, 0.997)	0.57 (0.311, 0.829)”	0.0020	0.0028
3	MTV, Shape-based PCs (1 to 3)	0.356 (0.144, 0.568)/0.883 (0.751, 1)	0.464 (0.329, 0.599)	0.0004	<0.0001
4	MTV, Shape-based PCs (1 to 3) and LDH	0.706 (0.503, 0.909)/0.828 (0.686, 0.97)	0.697 (0.509, 0.885)	<0.0001	<0.0001

D1. Logistic Regression using Shape-based PCs on PET Images (Extranodal group)					
	Variable	Sensitivity/Specificity	E[AUC]	Prognosis	
				OS (p-val)	PFS (p-val)
1	MTV (whole-body)	0.87 (0.751, 0.989)/0.692 (0.503, 0.881)	0.735 (0.584, 0.886)	0.0002	<0.0001
2	Shape-based PCs (PC 1 to 3)	0.701 (0.597, 0.805)/0.712 (0.478, 0.946)	0.675 (0.532, 0.818)	0.0180	0.0160
3	MTV, Shape-based PCs (1 to 3)	0.742 (0.493, 0.991)/0.847 (0.698, 0.996)	0.783 (0.7, 0.866)	<0.0001	<0.0001
4	MTV, Shape-based PCs (1 to 3) and LDH	0.704 (0.285, 1)/0.789 (0.577, 1)	0.682 (0.425, 0.939)	<0.0001	<0.0001

## Discussion

In this study, we developed and validated a quantitative radiological imaging signature derived from DLBCL PET/CT scans that could be used as a biomarker to predict response in patients treated with axi-cel treatment and allow us to identify patients that would benefit from the advanced immunotherapy treatment. We further divided the radiomic metrics into three functional characteristics related to Size, Shape, Texture, and computed PCs in each category. The ensemble feature signatures in each of the categories were used in a logistic regression model to predict treatment response (1 year). We identified the benefit of radiomic feature extraction for the prediction of durable survival in R/R/DLBCL. We found that the Shape-based PCs showed comparable performance to MTV (see **Table 2**). Furthermore, they continue to complement MTV for patients with a smaller tumor burden (≤ median MTV) (see **Supplementary Table S7**). It has been shown that size-based features, including MTV-related metrics, are often prognostic for a patient’s outcome, as previously reported by us and others (12, 13, 28). A few other studies have associated the shape of lesions with aggressive phenotypes and was shown to have poor prognosis in oncological diseases (3, 29, 30). In our study, we observed that shape-based predictors select patients with large irregularly featured nodules, while more round features are associated with low features that are associated with better prognostic cases (see **Figure 4**).

The current clinical consensus assessment criteria (Cheson and Lugano classifications) (22, 31) do not always accurately capture the disease condition nor can they predict disease outcome, creating a need to develop better biomarkers. Characterizing subtle physiological changes observed on radiological imaging using quantitative metrics has shown enormous promise in developing models that can be related to patient outcomes (14, 16, 32). Our metrics may be capable of capturing nuances across lymphatic and extranodal lesions. Most extranodal lymphoma lesions are restricted to a single organ but may have infiltrative bone involvement, making them multifocal (31, 33). It is interesting that radiologists usually assess PET/CT images by characterizing the intensity and lesion shape characteristics to discern true cancerous lesions from benign lesions, an essential step in assessing the disease burden or MTV in lymphoma. Specifically, it has been reported that normal lymph nodes are often elongated in shape and have uniform non-thickened nodal cortices and a preserved fatty hilum (31). Similar observations have been reported for related shape-based characteristics and their ability to predict outcomes in oncological diseases (32, 34).

Lymphomatous lesions may manifest in a variety of appearances but are typically classified based on their cellular architecture (follicular or diffuse) and morphology (small or large) (35). Beyond tumor metabolic activity, the shape of the lesions observed on PET/CT also plays a diagnostic role in lymphoma (36). There have been many studies assessing the role of axi-cel therapy in DLBCL through immune dysregulation (10), but the role of advanced imaging analytics has not been adequately characterized.

Most quantitative assessments in lymphoma utilize MTV, which has been shown to be prognostic (12, 37) but is still not clinically adopted. This metric provides a gross measurement of disease burden in a patient and does not assess nor discriminate based on the subtle characteristics of the lesions. Our study independently assessed lesion changes (on the pixel level) in a 3D region in FDG-PET images that were matched to corresponding CT regions, revealing subtle details with higher levels of contrast compared to PET imaging alone (23). Recently, MTV combined with LDH was associated with progression-free survival and the radiomic metrics of these patients were associated with a complete response (19). In predictive performance, these radiomic features demonstrated comparable predictive performance within our cohort (see **Table 2**, **Figure 2**).

Our finding provides a methodology for the use of radiological imaging-based quantitative metrics to assess patients’ response to immunotherapy (axi-cel) in DLBCL. Our systematic approach allows us to characterize the lesions’ shape irregularities and heterogeneity (including the microenvironment). It is well recognized that radiomic metrics are dependent on system-level variables such as scan parameters and image quality but despite these, the metrics can discriminate (38). Additionally, it is often difficult to explicitly relate a given radiomic feature to human observable characteristics, except for (mostly) size and shape metrics. Our study found that non-circular shaped lesions (high Shape PC) had a poor prognosis compared to close-to-circular-shaped lesions (low Shape PC) (see **Figure 4**).

It should be noted that shape-based metrics have been shown to have better reproducibility compared to texture metrics (39, 40). A recent study (20), found that a radiomic PET signature (using four features) achieved an AUC of 0.73 for predicting clinical benefit, outperforming metabolic tumor volume (AUC of 0.66).

Furthermore, our study needs secondary validation to provide prognostic benefits to patients treated with CAR-T for DLBCL. We retrospectively obtained diverse patient data for this study at our center and from a consortium that conducted the preliminary trial. Despite the efforts, the converged samples used in the study were limited, and we require additional independent samples to validate our findings. To mitigate and minimize study bias, we performed cross-validation to estimate predictor performance and reported our findings as an average (test) metric over multiple samplings. Our study provides several potential signatures (features) that need further validation to establish their use in the clinical setting and provide benefits for our patients. Our study follows the recommendations from the Standards for Reporting Diagnostic Accuracy Studies and Radiomics Quality Score to promote the reproducibility of clinical studies.

## Conclusion

This study evaluates the ability of radiological imaging characteristics obtained from PET/CT images to assess DLBCL patients’ treatment outcomes. The quantified shape-based radiomic features (and some texture-based features) observed on PET images predicted patient’s response to axi-cel (CAR-T) immunotherapy treatment and were comparable to metabolic tumor volume in this regard.

## Data Availability

The data analyzed in this study is subject to the following licenses/restrictions: Patient imaging data can be shared after de-identification and data transfer agreement with the institution. Requests to access these datasets should be directed to moffitt.org. The consortium patient cohort was previously published as part of the clinical trial (Zuma-1, NCT02348216). Request for data for consortium patients will be directed to the sponsor (Kite pharma®).
