# Supplementary material for: Radiomic features of PET/CT imaging of large B cell lymphoma lesions predicts CAR T cell therapy efficacy
Source: Front Oncol. 2024 Nov 25;14:1485039. doi: 10.3389/fonc.2024.1485039 (PMC11629080; doi:10.3389/fonc.2024.1485039)
Supplement: Supplementary file 1 [file DataSheet1.pdf]

SUPPLEMENTAL SECTION
FOR

Title: Radiomic Features on PET/CT Imaging of Large B cell Lymphoma Lesions Predicts CAR T-cell Therapy Efficacy

Authors:

Yoganand Balagurunathan[1,*], Ph.D., Zhouping Wei[1], PhD., Jin Qi, MD[2]., Zachary Thompson[3], Erin Dean[4,5], MD., Hong Lu[2,6], MD., Saran Vardhanabhuti[7], Salvatore Corallo[4]., Jung W. Choi[8], MD., Ph.D., Jenny J. Kim[6], MD, Mike Mattie[6], Ph.D., Michael Jain[4], MD., Frederick L. Locke[4], MD.

Department of [1] Machine Learning, [2] Cancer Physiology, [3] Biostatistics and Bioinformatics, [4] Blood and Marrow Transplant, [8] Diagnostic Imaging & Interventional Radiology, H. Lee. Moffitt Cancer Center, Tampa.

[5] University of Florida, USA.

[6] Tianjin Medical Hospital and Cancer, China.

[7] Kite, a Gilead Company, Santa Monica, CA

Running title: Radiomics features in DBCL patients treated with CAR-T therapy

***Corresponding Authors**: Drs. Balagurunathan/Locke,

Department of Machine Learning/ Bone Marrow Transplant,

H lee Moffitt Cancer Center, Tampa, FL.

Email: Yoganand.balagurunathan@moffitt.org, Frederick.locke@moffitt.org.

Keywords: Imaging biomarkers, radiomics, PET/CT scan, biomarkers in CAR-T cell therapy, lymphoma, radiomics in immunotherapy

Data Sharing Statement:

Patient's clinical data, along with deidentified imaging for our institutional patients, will be shared after the data transfer agreement. The consortium patient cohort was previously released as part of the clinical trial (Zuma-1, NCT02348216) and through a prior publication (DOI: 10.1056/NEJMoa170744). Request for imaging data for consortium patients will be directed to the sponsor (Kite pharma®).

Table S.1. Feature categories (3D Image features).

**Category**	**Description**	**Number of Descriptors**
C1: Size	Size (volume/size descriptors)	38
C2: Shape	Shape related	9
C3: Texture	Pixel Intensity -Histogram, Grayscale: Runlength & CoOccurrence, Texture: Laws & Wavelets	259
**Total**		**306**

Table S.2. Quantitative Image features used to describe the lesion of interest, computed independently in each image modality (CT/PET).

Sno		Description of the Features	Feature Category
1		F43:Volume_at_intensity_fraction_10	C1: Tumor Size
2		F44:Volume_at_intensity_fraction_90	
3		F45:Intensity_at_volume_fraction_10	
4		F46:Intensity_at_volume_fraction_90	
5		F47:Volume_at_intensity_fraction_difference	
6		F48:Intensity_at_volume_fraction_difference	
7		F50:Volume_(mm^3)	
8		F51:Approximate Volume_(mm^3)	
9		F52:Surface_area_(mm^2)	
10		F53:Surface_to_volume_ratio_(mm^2)	
11		F60:Longest_diameter(mm)	
12		F61:Major_axis_length	
13		F62:Minor_axis_length	
14		F63:Least_axis_length	
15		F64:Elongation	
16		F65:Flatness	
17		F66:Volume_density_axis-aligned_bounding_box	
18		F67:Area_density_axis_aligned_bounding_box	
19		F68:Volume_density_oriented_bounding_box	
20		F69:Area_density_oriented_bounding_box	
21		F70:Volume_density_approximate_enclosing_ellipsoid	
22		F71:Area_density_approximate_enclosing_ellipsoid	
23		F72:Volume_density_minimum_volume_enclosing_ellipsoid	
24		F73:Area_density_minimum_volume_enclosing_ellipsoid	
25		F74:Volume_density_convex_hull	
26		F75:Area_density_convex_hull	
27		F76:Number_of_connected_3D_components	
28		F80:CoM_x_(pxl)	
29		F81:CoM_y_(pxl)	
30		F82:CoM_z_(pxl)	
31		F83:CoM_x_(mm)	
32		F84:CoM_y_(mm)	
33		F85:CoM_z_(mm)	
34		F86:Weighted_CoM_x_(mm)	
35		F87:Weighted_CoM_y_(mm)	
36		F88:Weighted_CoM_z_(mm)	
37		F89:Border_length_(pxl)	
38		F90:Border_length_(mm)	
39		F54:Compactness_1	C2: Shape
40		F55:Compactness_2	
41		F56:Spherical_disproportion	
42		F57:Sphericity	
43		F58:Asphericity	
44		F59:Centre_of_mass_shift_(mm)	
45		F77:Asymmetry	
46		F78:Eccentricity	

47		F79:Orientation	
48		F1:Statistical_Mean	C3: Texture: (Pixel Intensity, Histogram, Grayscale - Runlength, CoOccurrence, Laws & Wavelets).
49		F2:Statistical_Variance	
50		F3:Statistical_SD	
51		F4:Statistical_SKEW	
52		F5:Statistical_Kurtosis	
53		F6:Statistical_Median	
54		F7:Statistical_Minimum_grey_level	
55		F8:Statistical_10th_percentile	
56		F9:Statistical_90th_percentile	
57		F10:Statistical_Maximum_grey_level	
58		F11:Statistical_Interquartile_range	
59		F12:Statistical_range	
60		F13:Statistical_mean_absolute_deviation	
61		F14:Statistical_Robust_mean_absolute_deviation	
62		F15:Statistical_Median_absolute_deviation	
63		F16:Statistical_Coefficient_of_variance	
64		F17:Statistical_Quartile_coefficient_of_dispersion	
65		F18:Statistical_ENERGY	
66		F19:Statistical_Root_mean_square	
67		F20:Intensity_histogram_mean	
68		F21:Intensity_histogram_variance	
69		F22:Intensity_histogram_skewness	
70		F23:Intensity_histogram_kurtosis	
71		F24:Intensity_histogram_median	
72		F25:Intensity_histogram_minimum_grey_level	
73		F26:Intensity_histogram_10th_percentile	
74		F27:Intensity_histogram_90th_percentile	
75		F28:Intensity_histogram_maximum_grey_level	
76		F29:Intensity_histogram mode	
77		F30:Intensity_histogram_interquartile_range	
78		F31:Intensity_histogram_range	
79		F32:Intensity_histogram_mean_absolute_deviation	
80		F33:Intensity_histogram_robust_mean_absolute_deviation	
81		F34:Intensity_histogram_median_absolute_deviation	
82		F35:Intensity_histogram_coefficient_of_variance	
83		F36:Intensity_histogram_quartile_coefficient_of_dispersion	
84		F37:Intensity_histogram entropy	
85		F38:Intensity_histogram_uniformity	
86		F39:Maximum_histogram_gradient	
87		F40:Maximum_histogram_gradient_grey_level	
88		F41:Minimum_histogram_gradient	
89		F42:Minimum_histogram_gradient_grey_level	Laws & Wavelet Texture (feature at different layers)
90		F92:avgCoocurrence_Joint_MAX	
91		F93:avgCoocurrence_Joint_Average	
92		F94:avgCoocurrence_Joint_variance	
93		F95:avgCoocurrence_Joint_entropy	
94		F96:avgCoocurrence_Difference_average	
95		F97:avgCoocurrence_Difference_variance	
96		F98:avgCoocurrence_Difference_entropy	
97		F99:avgCoocurrence_Sum_average	
98		F100:avgCoocurrence_Sum_variance	

99		F101:avgCoocurrence_Sum_entropy	
100		F102:avgCoocurrence_Angular_second_moment	
101		F103:avgCoocurrence_Contrast	
102		F104:avgCoocurrence_Dissimilarity	
103		F105:avgCoocurrence_Inverse_difference_(Homogeneity)	
104		F106:avgCoocurrence_Inverse_difference_normalized_(Homogeneity_normalized)	
105		F107:avgCoocurrence_Inverse_difference_moment	
106		F108:avgCoocurrence_Inverse_difference_moment_normalized	
107		F109:avgCoocurrence_Inverse_variance	
108		F110:avgCoocurrence_Correlation	
109		F111:avgCoocurrence_Autocorrelation	
110		F112:avgCoocurrence_Cluster_tendency	
111		F113:avgCoocurrence_Cluster_shade	
112		F114:avgCoocurrence_Cluster_prominence	
113		F115:avgCoocurrence_First_measure_of_information_correlation	
114		F116:avgCoocurrence_Second_measure_of_information_correlation	
115		F117:avg_3D_SRE_(Short_runs_emphasis)	
116		F118:avg_3D_LRE_(Long_runs_emphasis)	
117		F119:avg_3D_LGRE_(Low_grey_level_run_emphasis)	
118		F120:avg_3D_HGRE_(High_grey_level_run_emphasis)	
119		F121:avg_3D_SRLGE_(Short_run_low_grey_level_emphasis)	
120		F122:avg_3D_SRHGE_(Short_run_high_grey_level_emphasis)	
121		F123:avg_3D_LRLGE_(Long_run_low_grey_level_emphasis)	
122		F124:avg_3D_LRHGE_(Long_run_high_grey_level_emphasis)	
123		F125:avg_3D_GLN_(Grey_level_non_uniformity)	
124		F126:avg_3D_GLN_normalize_(Grey_level_non_uniformity_normalised)	
125		F127:avg_3D_RLN_(Run_length_non_uniformity)	
126		F128:avg_3D_RLN_normalize_(Run_length_non_uniformity_normalised)	
127		F129:avg_3D_RP_(Run_percentage)	
128		F130:avg_3D_GV_(Grey_level_variance)	
129		F131:avg_3D_RLV_(Run_length_variance)	
130		F132:avg_3D_RE_(Run_entropy)	
131		F133:GLSZM_Small_zone_emphasis	
132		F134:GLSZM_Large_zone_emphasis	
133		F135:GLSZM_Low_grey_level_zone_emphasis	
134		F136:GLSZM_High_grey_level_zone_emphasis	
135		F137:GLSZM_Small_zone_low_grey_level_emphasis	
136		F138:GLSZM_Small_zone_high_grey_level_emphasis	
137		F139:GLSZM_Large_zone_low_grey_level_emphasis	
138		F140:GLSZM_Large_zone_high_grey_level_emphasis	
139		F141:GLSZM_Grey_level_non_uniformity	
140		F142:GLSZM_Grey_level_non_uniformity_normalised	
141		F143:GLSZM_Zone_size_non_uniformity	
142		F144:GLSZM_Zone_size_non_uniformity_normalised	
143		F145:GLSZM_Zone_percentage	
144		F146:GLSZM_Grey_level_variance	
145		F147:GLSZM_Zone_size_variance	
146		F148:GLSZM_Zone_size_entropy	
147		F149:NGTDM_Coarseness	
148		F150:NGTDM_Contrast	
149		F151:NGTDM_Busyness	
150		F152:NGTDM_Complexity	
151		F153:NGTDM_Strength	

152		F154:3D_Laws_features_L5_L5_L5	
153		F155:3D_Laws_features_L5_L5_E5	
154		F156:3D_Laws_features_L5_L5_S5	
155		F157:3D_Laws_features_L5_L5_R5	
156		F158:3D_Laws_features_L5_L5_W5	
157		F159:3D_Laws_features_L5_E5_L5	
158		F160:3D_Laws_features_L5_E5_E5	
159		F161:3D_Laws_features_L5_E5_S5	
160		F162:3D_Laws_features_L5_E5_R5	
161		F163:3D_Laws_features_L5_E5_W5	
162		F164:3D_Laws_features_L5_S5_L5	Laws & Wavelet Texture<br><br>(feature at different layers)
163		F165:3D_Laws_features_L5_S5_E5	
164		F166:3D_Laws_features_L5_S5_S5	
165		F167:3D_Laws_features_L5_S5_R5	
166		F168:3D_Laws_features_L5_S5_W5	
167		F169:3D_Laws_features_L5_R5_L5	
168		F170:3D_Laws_features_L5_R5_E5	
169		F171:3D_Laws_features_L5_R5_S5	
170		F172:3D_Laws_features_L5_R5_R5	
171		F173:3D_Laws_features_L5_R5_W5	
172		F174:3D_Laws_features_L5_W5_L5	
173		F175:3D_Laws_features_L5_W5_E5	
174		F176:3D_Laws_features_L5_W5_S5	
175		F177:3D_Laws_features_L5_W5_R5	
176		F178:3D_Laws_features_L5_W5_W5	
177		F179:3D_Laws_features_E5_L5_L5	
178		F180:3D_Laws_features_E5_L5_E5	
179		F181:3D_Laws_features_E5_L5_S5	
180		F182:3D_Laws_features_E5_L5_R5	
181		F183:3D_Laws_features_E5_L5_W5	
182		F184:3D_Laws_features_E5_E5_L5	
183		F185:3D_Laws_features_E5_E5_E5	
184		F186:3D_Laws_features_E5_E5_S5	
185		F187:3D_Laws_features_E5_E5_R5	
186		F188:3D_Laws_features_E5_E5_W5	
187		F189:3D_Laws_features_E5_S5_L5	
188		F190:3D_Laws_features_E5_S5_E5	
189		F191:3D_Laws_features_E5_S5_S5	
190		F192:3D_Laws_features_E5_S5_R5	
191		F193:3D_Laws_features_E5_S5_W5	
192		F194:3D_Laws_features_E5_R5_L5	
193		F195:3D_Laws_features_E5_R5_E5	
194		F196:3D_Laws_features_E5_R5_S5	
195		F197:3D_Laws_features_E5_R5_R5	
196		F198:3D_Laws_features_E5_R5_W5	
197		F199:3D_Laws_features_E5_W5_L5	
198		F200:3D_Laws_features_E5_W5_E5	
199		F201:3D_Laws_features_E5_W5_S5	
200		F202:3D_Laws_features_E5_W5_R5	
201		F203:3D_Laws_features_E5_W5_W5	
202		F204:3D_Laws_features_S5_L5_L5	
203		F205:3D_Laws_features_S5_L5_E5	

204		F206:3D_Laws_features_S5_L5_S5	
205		F207:3D_Laws_features_S5_L5_R5	
206		F208:3D_Laws_features_S5_L5_W5	
207		F209:3D_Laws_features_S5_E5_L5	
208		F210:3D_Laws_features_S5_E5_E5	
209		F211:3D_Laws_features_S5_E5_S5	
210		F212:3D_Laws_features_S5_E5_R5	
211		F213:3D_Laws_features_S5_E5_W5	
212		F214:3D_Laws_features_S5_S5_L5	
213		F215:3D_Laws_features_S5_S5_E5	
214		F216:3D_Laws_features_S5_S5_S5	
215		F217:3D_Laws_features_S5_S5_R5	
216		F218:3D_Laws_features_S5_S5_W5	
217		F219:3D_Laws_features_S5_R5_L5	
218		F220:3D_Laws_features_S5_R5_E5	
219		F221:3D_Laws_features_S5_R5_S5	
220		F222:3D_Laws_features_S5_R5_R5	
221		F223:3D_Laws_features_S5_R5_W5	
222		F224:3D_Laws_features_S5_W5_L5	
223		F225:3D_Laws_features_S5_W5_E5	
224		F226:3D_Laws_features_S5_W5_S5	
225		F227:3D_Laws_features_S5_W5_R5	
226		F228:3D_Laws_features_S5_W5_W5	
227		F229:3D_Laws_features_R5_L5_L5	
228		F230:3D_Laws_features_R5_L5_E5	
229		F231:3D_Laws_features_R5_L5_S5	
230		F232:3D_Laws_features_R5_L5_R5	
231		F233:3D_Laws_features_R5_L5_W5	
232		F234:3D_Laws_features_R5_E5_L5	
233		F235:3D_Laws_features_R5_E5_E5	
234		F236:3D_Laws_features_R5_E5_S5	
235		F237:3D_Laws_features_R5_E5_R5	
236		F238:3D_Laws_features_R5_E5_W5	
237		F239:3D_Laws_features_R5_S5_L5	
238		F240:3D_Laws_features_R5_S5_E5	
239		F241:3D_Laws_features_R5_S5_S5	
240		F242:3D_Laws_features_R5_S5_R5	
241		F243:3D_Laws_features_R5_S5_W5	
242		F244:3D_Laws_features_R5_R5_L5	
243		F245:3D_Laws_features_R5_R5_E5	
244		F246:3D_Laws_features_R5_R5_S5	
245		F247:3D_Laws_features_R5_R5_R5	
246		F248:3D_Laws_features_R5_R5_W5	
247		F249:3D_Laws_features_R5_W5_L5	
248		F250:3D_Laws_features_R5_W5_E5	
249		F251:3D_Laws_features_R5_W5_S5	
250		F252:3D_Laws_features_R5_W5_R5	
251		F253:3D_Laws_features_R5_W5_W5	
252		F254:3D_Laws_features_W5_L5_L5	
253		F255:3D_Laws_features_W5_L5_E5	
254		F256:3D_Laws_features_W5_L5_S5	
255		F257:3D_Laws_features_W5_L5_R5	
256		F258:3D_Laws_features_W5_L5_W5	

257		F259:3D_Laws_features_W5_E5_L5	
258		F260:3D_Laws_features_W5_E5_E5	
259		F261:3D_Laws_features_W5_E5_S5	
260		F262:3D_Laws_features_W5_E5_R5	
261		F263:3D_Laws_features_W5_E5_W5	
262		F264:3D_Laws_features_W5_S5_L5	
263		F265:3D_Laws_features_W5_S5_E5	
264		F266:3D_Laws_features_W5_S5_S5	
265		F267:3D_Laws_features_W5_S5_R5	
266		F268:3D_Laws_features_W5_S5_W5	
267		F269:3D_Laws_features_W5_R5_L5	
268		F270:3D_Laws_features_W5_R5_E5	
269		F271:3D_Laws_features_W5_R5_S5	
270		F272:3D_Laws_features_W5_R5_R5	
271		F273:3D_Laws_features_W5_R5_W5	
272		F274:3D_Laws_features_W5_W5_L5	
273		F275:3D_Laws_features_W5_W5_E5	
274		F276:3D_Laws_features_W5_W5_S5	
275		F277:3D_Laws_features_W5_W5_R5	
276		F278:3D_Laws_features_W5_W5_W5	
277		F279:3D_Wavelet_P1_L2_C1	
278		F280:3D_Wavelet_P2_L2_C1	
279		F281:3D_Wavelet_P1_L2_C2	
280		F282:3D_Wavelet_P2_L2_C2	
281		F283:3D_Wavelet_P1_L2_C3	
282		F284:3D_Wavelet_P2_L2_C3	
283		F285:3D_Wavelet_P1_L2_C4	
284		F286:3D_Wavelet_P2_L2_C4	
285		F287:3D_Wavelet_P1_L2_C5	
286		F288:3D_Wavelet_P2_L2_C5	
287		F289:3D_Wavelet_P1_L2_C6	
288		F290:3D_Wavelet_P2_L2_C6	
289		F291:3D_Wavelet_P1_L2_C7	
290		F292:3D_Wavelet_P2_L2_C7	
291		F293:3D_Wavelet_P1_L2_C8	
292		F294:3D_Wavelet_P2_L2_C8	
293		F295:3D_Wavelet_P1_L2_C9	
294		F296:3D_Wavelet_P2_L2_C9	
295		F297:3D_Wavelet_P1_L2_C10	
296		F298:3D_Wavelet_P2_L2_C10	
297		F299:3D_Wavelet_P1_L2_C11	
298		F300:3D_Wavelet_P2_L2_C11	
299		F301:3D_Wavelet_P1_L2_C12	
300		F302:3D_Wavelet_P2_L2_C12	
301		F303:3D_Wavelet_P1_L2_C13	
302		F304:3D_Wavelet_P2_L2_C13	
303		F305:3D_Wavelet_P1_L2_C14	
304		F306:3D_Wavelet_P2_L2_C14	
305		F307:3D_Wavelet_P1_L2_C15	
306		F308:3D_Wavelet_P2_L2_C15	

Table S.3. Description of Texture Features

A. **Run-length analysis:** Run-length texture features [1] examine runs of similar gray values in an image. Runs may be labeled according to length, gray value, and direction (horizontal or vertical). Long runs of the same gray value correspond to coarser textures, whereas shorter runs correspond to finer textures. In our study, texture information was quantified by computing 11 features [2] derived from the run-length distribution matrix. They are: 1: Short Run Emphasis (SRE). 2: Long Run Emphasis (LRE). 3: Gray-Level Non-uniformity (GLN). 4: Run Length Non-uniformity (RLN). 5: Run Percentage (RP). 6: Low Gray-Level Run Emphasis (LGRE). 7: High Gray-Level Run Emphasis (HGRE). 8: Short Run Low Gray-Level Emphasis (SRLGE). 9: Short Run High Gray-Level Emphasis (SRHGE). 10: Long Run Low Gray-Level Emphasis (LRLGE). 11: Long Run High Gray-Level Emphasis (LGHGE).

Let $p(i,j)$ be the element of run-length matrix, let M be the number of gray levels, N be the maximum run length. n_r is the total number of runs, n_p is the number of pixels in the image. Define 3 new matrices first.

(a) $p_p(i,j) = p(i,j) * j$

(b) $p_g(i) = \sum_{j=1}^{N} p(i,j)$

(c) $p_r(j) = \sum_{i=1}^{M} p(i,j)$

1. Short Run Emphasis (SRE). $SRE=\frac{1}{n_r}\sum_{j=1}^{N}\frac{p_r(j)}{j^2}$
2. Long Run Emphasis (LRE). $LRE=\frac{1}{n_r}\sum_{j=1}^{N}p_r(j) * j^2$
3. Gray-Level Nonuniformity. $GLN=\frac{1}{n_r}\sum_{i=1}^{M}p_g(i)^2$
4. Run Length Nonuniformity. $RLN=\frac{1}{n_r}\sum_{j=1}^{N}p_r(j)^2$
5. Run Percentage. $RP=\frac{n_r}{n_p}$
6. Low Gray-Level Run Emphasis. $LGRE=\frac{1}{n_r}\sum_{i=1}^{M}\frac{p_g(i)}{i^2}$
7. High Gray-Level Run Emphasis. $HGRE=\frac{1}{n_r}\sum_{i=1}^{M}p_g(i) * i^2$

8. Short Run Low Gray-Level Emphasis.

$SRLGE=\frac{1}{n_r}\sum_{i=1}^{M}\sum_{j=1}^{N}\frac{p(i,j)}{i^2 * j^2}$

9. Short Run High Gray-Level Emphasis.

$SRHGE=\frac{1}{n_r}\sum_{i=1}^{M}\sum_{j=1}^{N}\frac{p(i,j)*i^2}{j^2}$

10. Long Run Low Gray-Level Emphasis.

$LRLGE=\frac{1}{n_r}\sum_{i=1}^{M}\sum_{j=1}^{N}\frac{p(i,j)*j^2}{i^2}$

11. Long Run High Gray-Level Emphasis.

$LRHGE=\frac{1}{n_r}\sum_{i=1}^{M}\sum_{j=1}^{N}p(i,j) * i^2 * j^2$

The Co-occurrence matrices and run-length analysis features can be obtained in 3D [3], the features are calculated in 13 different directions; with each direction, the processing is done by plane instead of slice. Hence, information between slices is not ignored.

B. **Co-occurrence matrices:** the co-occurrence matrix [4] is a matrix that contains the frequency of one gray level intensity appearing in a specified spatial linear relationship with another gray level intensity within a certain range. Computation of features requires first constructing the co-occurrence matrix, then different measurements [5] The matrix can be used to calculate the measurements, which include contrast, energy, homogeneity, entropy, mean, and max probability.

Let $p(i,j)$ be the element of the co-occurrence matrix.

1. Contrast=$\sum_{i,j}|i-j|^2 * p(i,j)$
2. Energy=$\sum_{i,j}p(i,j) * p(i,j)$
3. Homogeneity=$\sum_{i,j}\frac{p(i,j)}{1+|i-j|}$
4. Entropy=$-\sum_{i,j}p(i,j) * \log(p(i,j))$
5. Sum Mean=0.5*$\sum_{i,j}(i+j) * p(i,j)$

Max probability=max($p(i,j)$).

C. **Laws features :** Laws features [6] were constructed from five one-dimensional filters, each designed to reflect a different type of structure in the image. These one-dimensional filters are defined as E5 (edges), S5 (spots), R5 (ripples), W5 (waves), and L5 (low pass or average gray value). Using these 1-D convolution filters, 2-D filters are generated by convolving pairs of these filters, such as L5L5, E5L5, S5L5, W5L5, R5L5, etc. We can generate 25 different 2-D filters. 3D laws filters were constructed similarly to 2D. 3D filters are generated by convolving 3 types of 1D filter, such as L5L5L5, L5L5E5, L5L5S5, L5L5R5, L5L5W5, etc. The total number of 3-D filters is 125. For the 3D case, after the convolution with the 3D filters for the image, the energy [7] of the texture feature was computed by the following equation:

$$Energy = \frac{1}{R}\sum_{i=N+1}^{I-N}\sum_{j=N+1}^{J-N}\sum_{k=N+1}^{K-N} h^2(i,j,k)$$,

Where R is a normalizing factor, I and J, K are image dimensions, h(i,j,k) is derived from the convolution filters and original image. For the 2D case, the above equation is very similar but without the 3rd (z direction) dimension.

D. Wavelet Decomposition:

The discrete wavelet transform [8] can iteratively decompose an image (2D) into four components. Each iteration splits the image horizontally and vertically into low-frequency (low pass) and high-frequency (high pass) components. Thus, four components are generated: a high-pass/high-pass component consisting of mostly diagonal structure, a high-pass/low-pass component consisting mostly of vertical structures, a low-pass/high-pass component consisting mostly of horizontal structure, and a low-pass/low-pass component that represents a blurred version of the original image. Subsequent iterations then repeat the decomposition on the low-pass/low-pass component from the previous iteration. These subsequent iterations highlight broader diagonal, vertical, and horizontal textures. And for each component, we calculated the energy (referred to with a suffix P1) & entropy (referred to with a suffix P2) feature. A wavelet transform of a 3D signal can be achieved by applying the 1D wavelet transform along all the three directions (x,y,z). Featured obtained in each level of

decomposition is referred with suffix L (example: L1, L2) and level of decomposition is referred to with a prefix C (example: C1 to C9).

$$Energy = \frac{1}{MxNxL}\sum_{i=1}^{M}\sum_{j=1}^{N}\sum_{k=1}^{L} I^2(i,j,k)$$

$$Entropy = \frac{-1}{MxNxL}\sum_{i=1}^{M}\sum_{j=1}^{N}\sum_{k=1}^{L}\left(\frac{I^2(i,j,k)}{norm^2}\right)\log\left(\frac{I^2(i,j,k)}{norm^2}\right)$$

I(i,j,k) shows the subblock elements and M, N, and L are the dimensions of each subblock and

$$norm^2 = \sum_i \sum_j \sum_k I^2(i,j,k)$$

The number of features really depends on the number of decomposition levels selected. 1 level: 2* 8(block) = 16 features, In 2 levels: 2* 15(block) = 30 features

E. Pixel Histogram Features: the pixel intensity histogram h(a) is the number of pixels that occurred for brightness level "a" plotted against their brightness level. The probability distribution of the brightness Prob(a) can also be calculated. Six features: mean, standard deviation, skewness, kurtosis, energy, and entropy were then incorporated.

$$\text{mean}=\sum_{i=1}^{range} i * prob(i)$$

$$\text{sd}=\sqrt{\sum_{i=1}^{range}(i-mean)^2 * prob(i)}$$

$$\text{skewness}=\frac{\sum_{i=1}^{range}(i-mean)^3 * prob(i)}{(\sum_{i=1}^{range}(i-mean)^2 * prob(i))^{1.5}}$$

$$\text{kurtosis}=\frac{\sum_{i=1}^{range}(i-mean)^4 * prob(i)}{(\sum_{i=1}^{range}(i-mean)^2 * prob(i))^2}$$

$$\text{energy}=\sum_{i=1}^{range} prob(i) * prob(i)$$

$$\text{entropy}=-\sum_{i=1}^{range} prob(i) * Log\,(prob(i))$$

Where intensity range is [0,range] (normalized).

F. Tumor Shape & Size: In addition to direct size (Univariate, bivariate) and volume measurements, various surrounding tumor parenchyma are measured; some of such measurements are described below.

Border Length:
The border length of a 3D image object is the sum of the border lengths of all image object slices multiplied by the spatial distance between the slices.

$$b_v = \left(\sum_{n=1}^{\#(\text{slices})} b_v\,(\text{Slice}) \right) * u_{\text{slices}} + b_v(Z)$$

Where, b_v: border length of image object v, b_v(slice): border length of image object slice, b_v(z): border length of the image object in the z-direction, u_{slices}: spatial distance between slices in the coordinate system unit.

Asymmetry:
The asymmetry (Asy) is calculated from the ratio between the smallest and largest eigenvalues of the image object.

$$\text{Asy} = 1 - \frac{\sqrt{\lambda_{min}}}{\sqrt{\lambda_{max}}}$$

Where λmin is the minimal eigenvalue and λmax is the maximal eigenvalue.

Compactness: The compactness (Comp) of a 3D image object is calculated by a scaled product of its three eigenvalues 2∗λ1, 2∗λ2, 2∗λ3 divided by the number of its pixel/voxel.

Comp = 2 λ1*2 λ2*2 λ3/Vv

Where, λ1: eigenvalue 1 of a 3D image object v, λ2: eigenvalue 2 of a 3D image object v, λ3: eigenvalue 3 of a 3D image object v, Vv: volume of image object v.

Density:
The Density(D) feature describes the spatial distribution of the pixels of an Image object. The ideal compact shape on a pixel raster is the cube. The more the shape of an image object is like a cube, the higher its density

$$D = \frac{\sqrt[3]{V_v}}{\sqrt{Var(X)+Var(Y)+Var(Z)}}$$

Where, Vv: volume of image object v, 3√V: edge of the volume fitted cube, √Var(X) + Var(Y) + Var (Z): radius of the fitted sphere

Roundness: Describes how similar the shape of an image object is to an ellipsoid. The more the shape of an image object is similar to an ellipsoid, the lower its roundness. It is calculated by the difference between the enclosing ellipsoid and the enclosed ellipsoid.

$$Roundness = \varepsilon_v^{max} - \varepsilon_v^{min}$$

Where εv (max) is the radius of the smallest enclosing ellipsoid
Ev (min) is the radius of the largest enclosed ellipsoid

Supplemental Table S4. Relationship between total body metabolic tumor volume (MTV) and Radiomics features PC (principal components) assessed using correlation coefficient. The top four correlated (in magnitude) principal components with MTV, repeated in both imaging modality (CT, PET) and category based on lymphatics/extranodal sites.

	**Radiomics PC**	**Spearman Correlation (ρ)**	**P-value**
Lymphatics (CT Images)			
	texturePC1	0.35	<0.01
	shapePC1	0.30	<0.01
	texturePC14	0.25	<0.01
	shapePC2	-0.22	0.014
Extra-Nodal (CT Images)			
	texturePC1	0.38	0.002
	texturePC52	-0.31	0.012
	texturePC44	-0.29	0.018
	texturePC49	-0.28	0.0216
Lymphatics (PET Images)			
	texturePC7	-0.33	<0.01
	texturePC10	-0.29	<0.01
	shapePC1	0.27	0.002
	texturePC5	-0.27	0.003
Extra-Nodal (PET Images)			
	shapePC1	0.36	0.0004
	texturePC3	0.34	0.0008
	texturePC94	0.26	0.0072
	texturePC1	-0.28	0.0073

Supplemental Table.S5. Cox regression model to assess the role of Metabolic tumor volume and Radiomics features to assess risk to over-all survival (OS) using the largest nodule in a patient scans that are categorized by: a) Lymphatics – CT, b) Lymphatic – PET, c) Extra-Nodal -CT, d) Extra-Nodal -PET, observed across the cohort.

a1.Cox Model (OS) – MTV, Radiomics, Clinical - CT (Lymphatic)			
	**Variables**	**Hazard Ratio**	**P-value**
	MTV	1.173 [1.067,1.29]	0.00099*
	LDH	1.00 [0.999,1.001]	0.6704
	Texture PC1	0.999 [0.932,1.07]	0.96895
	Shape PC1	1.018 [0.806,1.285]	0.88256
	Texture PC1: Shape PC1	0.994 [0.971,1.017]	0.60156

b1.Cox Model (OS) – MTV, Radiomics & Clinical on PET Images (Lymphatics)			
	**Variables**	**Hazard Ratio**	**P-value**
	MTV	1.202 [1.077,1.341]	0.00104*
	LDH	1.00 [0.999,1.001]	0.59649
	Texture PC1	1.024 [0.964,1.088]	0.43701
	Shape PC1	1.029 [0.86,1.231]	0.75385
	Texture PC1: Shape PC1	1.018 [0.995,1.041]	0.12498

c1.Cox Model (OS) – MTV, Radiomics & Clinical - CT (Extra-nodal)			
	**Variables**	**Hazard Ratio**	**P-value**
	MTV	1.142 [1.029,1.269]	0.01291*
	LDH	1.00 [0.999,1.002]	0.54026
	Texture PC1	0.999 [0.919,1.086]	0.98241
	Shape PC1	0.959 [0.689,1.335]	0.80577
	Texture PC1: Shape PC1	0.99 [0.961,1.02]	0.50609

d1. Cox Model (OS) – MTV, Radiomics & Clinical on PET Images (Extra-nodal)			
	**Variables**	**Hazard Ratio**	**P-value**
	MTV	1.183 [1.064,1.315]	0.00184*
	LDH	1.00 [0.999,1.001]	0.7837
	Texture PC1	0.968 [0.898,1.045]	0.40542
	Shape PC1	1.218 [1.048,1.416]	0.01011*
	Texture PC1: Shape PC1	0.986 [0.949,1.025]	0.48749

Supplemental Table .S6. Cox regression model to assess the role of Metabolic tumor volume and Radiomics features to assess risk to Progression free survival (PFS) using the largest nodule in a patient image scans that are categorized by: a) Lymphatics – CT, b) Lymphatic – PET, c) Extra-Nodal -CT, d) Extra-Nodal -PET, observed across the cohort.

a1. Cox Model (PFS) – MTV, Radiomics &Clinical on CT Images (Lymphatics)			
	**Variable**	**Hazard Ratio**	**P-value**
	MTV	1.153 [1.038,1.281]	0.00773*
	LDH	1.00 [0.999,1.001]	0.76614
	Texture PC1	1.006 [0.945,1.071]	0.85513
	Shape PC1	1.014 [0.817,1.258]	0.89998
	Texture PC1: Shape PC1	0.985 [0.963,1.008]	0.19832

b1. Cox Model (PFS) – MTV, Radiomics & Clinical on PET Images (Lymphatics)			
	**Variable**	**Hazard Ratio**	**P-value**
	MTV	1.138 [1.02,1.269]	0.02091*
	LDH	1.00 [0.999,1.001]	0.99799
	Texture PC1	1.035 [0.975,1.099]	0.26406
	Shape PC1	1.074 [0.905,1.275]	0.41429
	Texture PC1: Shape PC1	1.022 [0.999,1.046]	0.0611

c1. Cox Model (PFS) – MTV, Radiomics & Clinical on CT Images (Extra-nodal)			
	**Variable**	**Hazard Ratio**	**P-value**
	MTV	1.129 [1.012,1.258]	0.02958
	LDH	1.00 [0.999,1.001]	0.62081
	Texture PC1	1.028 [0.947,1.117]	0.51134
	Shape PC1	0.848 [0.609,1.181]	0.32987
	Texture PC1: Shape PC1	0.995 [0.966,1.024]	0.72324

d1. Cox Model (PFS) – MTV, Radiomics & Clinical on PET Images (Extra-nodal)			
	**Variable**	**Hazard Ratio**	**P-value**
	MTV	1.179 [1.059,1.313]	0.00267*
	LDH	0.999 [0.998,1.001]	0.34195
	Texture PC1	0.931 [0.85,1.02]	0.12681
	Shape PC1	1.181 [1.018,1.371]	0.0284*
	Texture PC1: Shape PC1	0.962 [0.925,1.002]	0.05976

Supplemental Table S.7. Logistic regression model to predict 1-year response to CAR T therapy using total body Metabolic tumor volume and Radiomic PC (Shape PC) in patient image scans that are associated with Extra-Nodal -PET. Patients are divided into cohorts based on their overall MTV: a) lower median (n=47) and b) upper median (n=47). Estimates were obtained using 5-fold cross validation cross-validation.

a. Logistic Regression using Shape based (Radiomics) on PET Images (Extra-Nodal): Lower MTV (n=47)					
	**Variable**	**Sensitivity/Specificity**	**E[AUC]**	**Prognosis**	
				**OS** (p-val)	**PFS** (p-val)
1	MTV *(total body)*	0.767 (0.601, 0.933)/0.871 (0.717, 1)	0.754 (0.537, 0.971)	0.0300	0.0007
2	Shape PCs (PC1 to 3)	0.84 (0.55, 1)/0.74 (0.5, 0.98)	0.716 (0.478, 0.954)	0.0770	0.0360
3	MTV, Shape PCs (1 to3)	0.633 (0.329, 0.937)/0.975 (0.913, 1)	0.67 (0.406, 0.934)	0.0860	0.0093
4	MTV, Shape PCs (1 to3) & LDH	0.717 (0.373, 1)/0.769 (0.501, 1)	0.601 (0.374, 0.828)	0.0011	0.0006

b.Logistic Regression using Shape based (Radiomics) on PET Images (Extra-Nodal): Higher MTV (n=47)					
	**Variable**	**Sensitivity/Specificity**	**E[AUC]**	**Prognosis**	
				**OS** (p-val)	**PFS** (p-val)
1	MTV *(total body)*	0.557 (0.365, 0.749)/0.85 (0.602, 1)	0.5 (0.395, 0.605)	0.16	0.42
2	Shape PCs (PC1 to 3)	0.584 (0.379, 0.789)/0.9 (0.652, 1)	0.547 (0.342, 0.752)	.5000	0.6200
3	MTV, Shape PCs (1 to3)	0.367 (0.187, 0.547)/0.9 (0.652, 1)	0.373 (0.176, 0.57)	0.3900	0.2300
4	MTV, Shape PCs (1 to3) & LDH	0.417 (0.105, 0.729)/0.933 (0.767, 1)	0.399 (0.132, 0.666)	0.3900	0.2300

Supplemental Table S.8. Logistic regression model to predict 1-year response to CAR T therapy using Metabolic tumor volume and Texture based (Radiomics) features in patients image scans that are associated with a) Lymphatics – CT, b) Lymphatic -PET, c) Extra-Nodal – CT, d) Extra-Nodal -PET, observed across the population. Estimates obtained using 5-fold cross-validation.

a1. Logistic Model using Texture based (Radiomics) on CT Images (Lymphatics)

	Variable	Sensitivity/Specificity	E[AUC]	Prognosis	
				**OS** (p-val)	**PFS(p-val)**
1	MTV *(total body)*	0.737 (0.608, 0.866)/0.737 (0.531, 0.943)	0.719 (0.572, 0.866)	<0.0001	0.0006
2	Texture PCs (1 to 3)	0.573 (0.356, 0.79)/0.691 (0.417, 0.965)	0.565 (0.482, 0.648)	0.5100	0.8100
3	MTV with Texture PCs (1 to3)	0.666 (0.349, 0.983)/0.774 (0.574, 0.974)	0.707 (0.597, 0.817)	<0.0001	0.0008
4	MTV with Texture PCs (1 to3) & LDH (real)	0.75 (0.514, 0.986)/0.699 (0.528, 0.87)"	0.678 (0.564, 0.792)	0.0003	0.0023

b1. Logistic Model using Texture based (Radiomics) on PET Images (Lymphatics)

	Variable	Sensitivity/Specificity	E[AUC]	Prognosis	
				**OS** (p-val)	**PFS(p-val)**
1	MTV *(total body)*	0.847 (0.689, 1)/0.681 (0.427, 0.935)	0.744 (0.581, 0.907)	0.0002	0.0026
2	Texture PCs (PC1 to 3)	0.831 (0.519, 1)/0.308 (0, 0.668)	0.461 (0.409, 0.513)	0.3900	0.9900
3	MTV, Texture PCs (1 to3)	0.658 (0.324, 0.992)/0.744 (0.616, 0.872)	0.631 (0.492, 0.77)	<0.0001	<0.0001
4	MTV, Texture PCs (1 to3) & LDH (real)	0.749 (0.625, 0.873)/0.653 (0.554, 0.752)	0.638 (0.537, 0.739)	0.0140	0.0560

c1. Logistic Model using Texture based (Radiomics) on CT Images (Extra-Nodal)

	Variable	Sensitivity/Specificity	E[AUC]	Prognosis	
				**OS** (p-val)	**PFS(p-val)**
1	MTV *(total body)*	0.709 (0.406, 1)/0.88 (0.715, 1)	0.758 (0.622, 0.894)	<0.0001	0.0018
2	Texture PCs (PC1 to 3)	0.779 (0.409, 1)/0.693 (0.424, 0.962)	0.655 (0.477, 0.833)	0.0015	0.0022
3	MTV, Texture PCs (1 to3)	0.818 (0.624, 1)/0.576 (0.32, 0.832)	0.613 (0.361, 0.865)	<0.0001	<0.0001
4	MTV, Texture PCs (1 to3) & LDH (real)	0.489 (0.122, 0.856)/0.819 (0.638, 1)	0.552 (0.291, 0.813)	<0.0001	0.0020

d1. Logistic Model using Texture based (Radiomics) on PET Images (Extra-Nodal)

	Variable	Sensitivity/Specificity	E[AUC]	Prognosis	
				**OS** (p-val)	**OS** (p-val)
1	MTV *(total body)*	0.87 (0.751, 0.989)/0.692 (0.503, 0.881)	0.735 (0.584, 0.886)	0.0003	<0.0001
2	Texture PCs (PC1 to 3)	0.649 (0.315, 0.983)/0.74 (0.413, 1)	0.589 (0.349, 0.829)	0.0043	0.0110
3	MTV, Texture PCs (1 to3)	0.679 (0.373, 0.985)/0.742 (0.537, 0.947)	0.655 (0.535, 0.775)	0.0001	0.0004
4	MTV, Texture PCs (1 to3) & LDH (real)	0.87 (0.751, 0.989)/0.692 (0.503, 0.881)	0.735 (0.584, 0.886)	0.0018	0.0012

Supplemental Table S.9. Logistic regression model to predict 1-year response to CAR T therapy using Metabolic tumor volume and Radiomic PC (Texture PC) in patient image scans that are associated with Extra-Nodal -PET. Patients were divided into cohorts based on their overall MTV, a) lower median (n=47) and b) upper median (n=47). Estimates obtained using 5-fold cross-validation.

a. *Lower MTV (below median)*

a. Logistic Regression using Texture based (Radiomics) on PET Images (Extra-Nodal): Lower median of MTV (n=47)			
	**Variable**	**Sensitivity/Specificity**	**E[AUC]**
1	MTV *(total body)*	0.77/0.87	0.75
2	Texture PCs (PC1 to 3)	0.84/0.62	0.64
3	MTV, Texture PCs (1 to3)	0.9/0.61	0.663
4	MTV, Texture PCs (1 to3) & LDH (real)	*0.58/0.83*	*0.58*

a. *Higher MTV (above median)*

b.Logistic Regression using Texture based (Radiomics) on PET Images (Extra-Nodal): Upper median of MTV (n=47)			
	**Variable**	**Sensitivity/Specificity**	**E[AUC]**
1	MTV *(total body)*	0.56/0.85	0.50
2	Texture PCs (PC1 to 3)	0.81/0.67	0.55
3	MTV, Texture PCs (1 to3)	0.61/0.8	0.56
4	MTV, Texture PCs (1 to3) & LDH (real)	0.41/1	0.49

Supplemental Figure SF.1. Process flow to develop quantitative radiological image-based biomarkers (Radiomics) and compare with gross tumor measurements (Metabolic Tumor Volume or MTV).

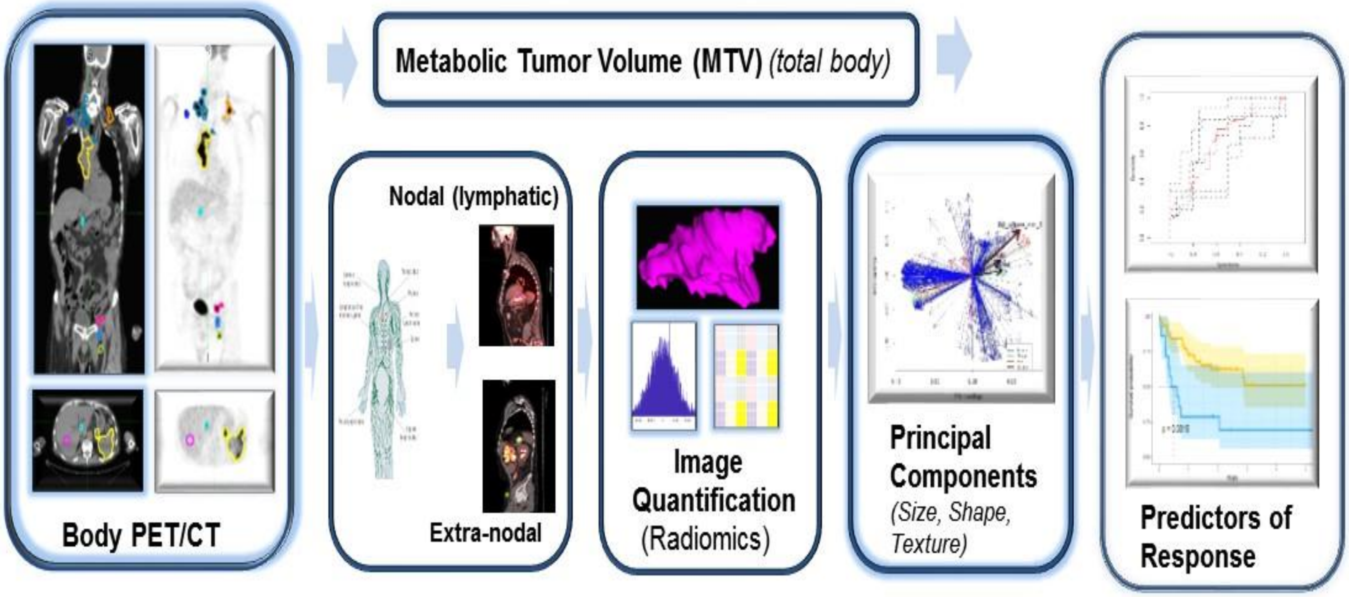

Supplemental Figure SF2. Scatter plots of Principal Components (PC1 &2) loading across categories of radiomic features (Size, Shape, Texture) with respect to total body metabolic tumor volume (MTV) and largest lesions volume (in mm³). A) Extra-Nodal CT features b) Extra-nodal PET (SUV) features.

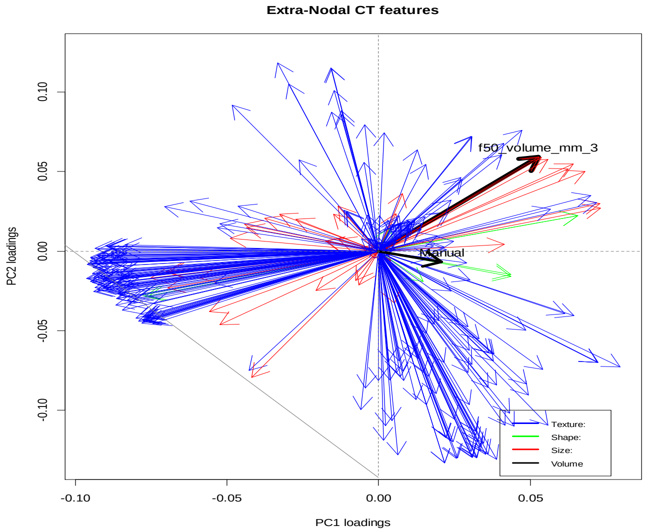

(A)

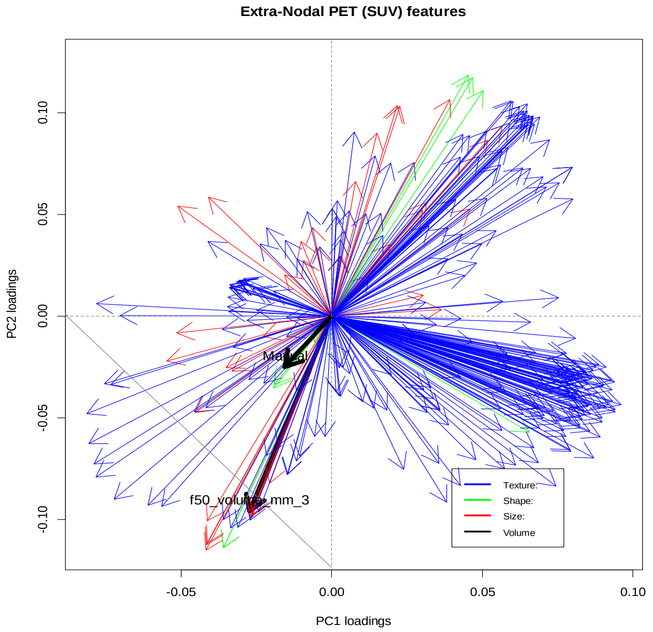

(B)

Supplemental Figure SF.3. Receiver operating characteristic curve to predict 1-year Overall survival, using a Logistic model based on texture features of the largest lesion in the Extra-nodal regions of PET (SUV) images (See **Suppl.ST. 4**). Models with MTV (average AUC 0.74), Texture based radiomic features (Principal components 1 to 3) (average AUC 0.59), Combined MTV with Texture based radiomic features (Principal components 1 to 3) (average AUC 0.66).

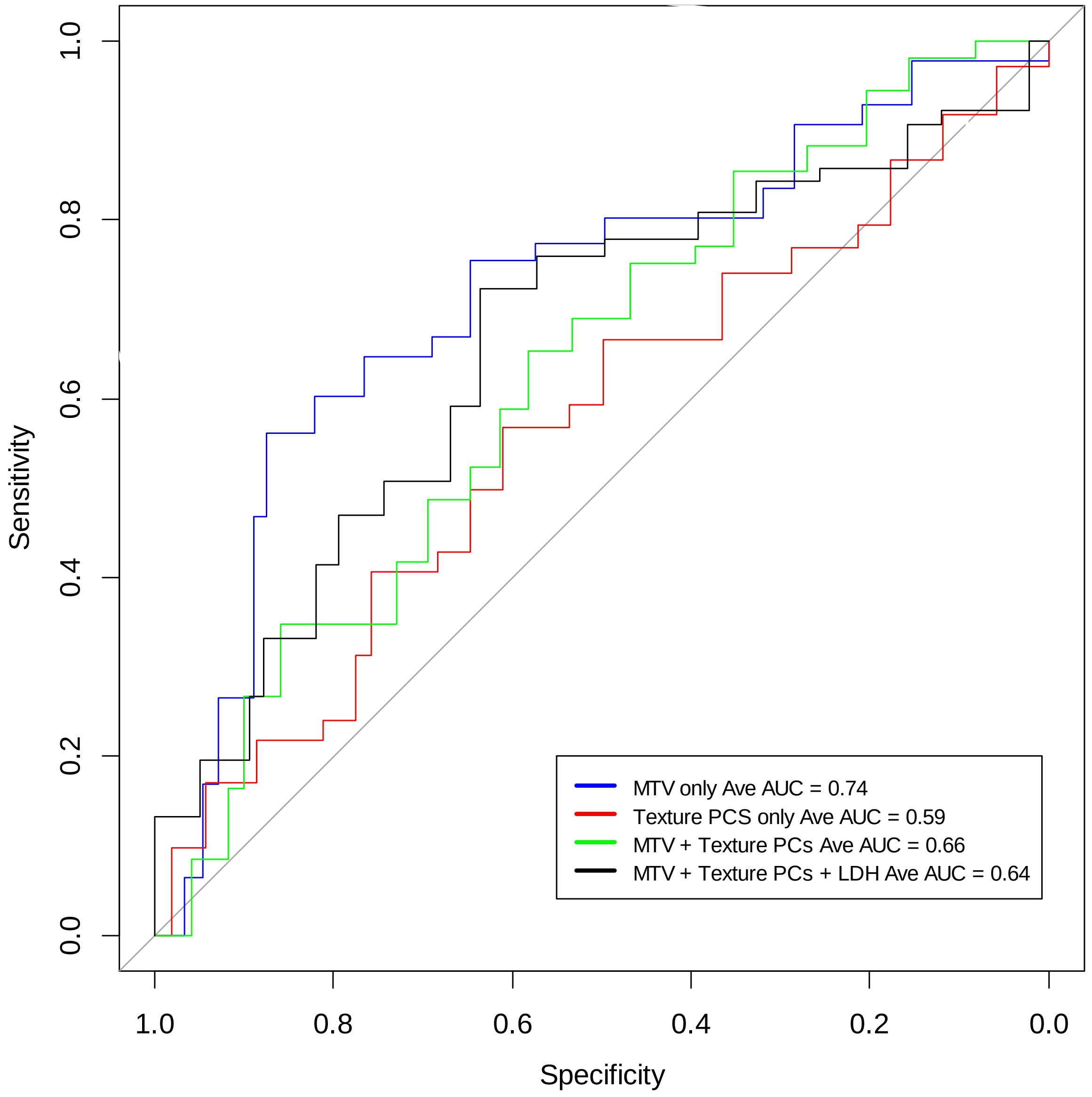

Supplemental Figure SF.4. Kaplan Meier (KM) obtained using patients grouped with a cut point obtained from a logistic model on <u>Shape based radiomic features (Principal components 1 to 3) extracted on Extra-nodal regions</u> of PET (SUV) images (details in Table 6); a) Overall survival, b) Progression free survival. Compared with logistic model-based cut point obtained using metabolic tumor volume (MTV) for c) OS and d) PFS.

(subplot: a & b are referenced in the main paper)

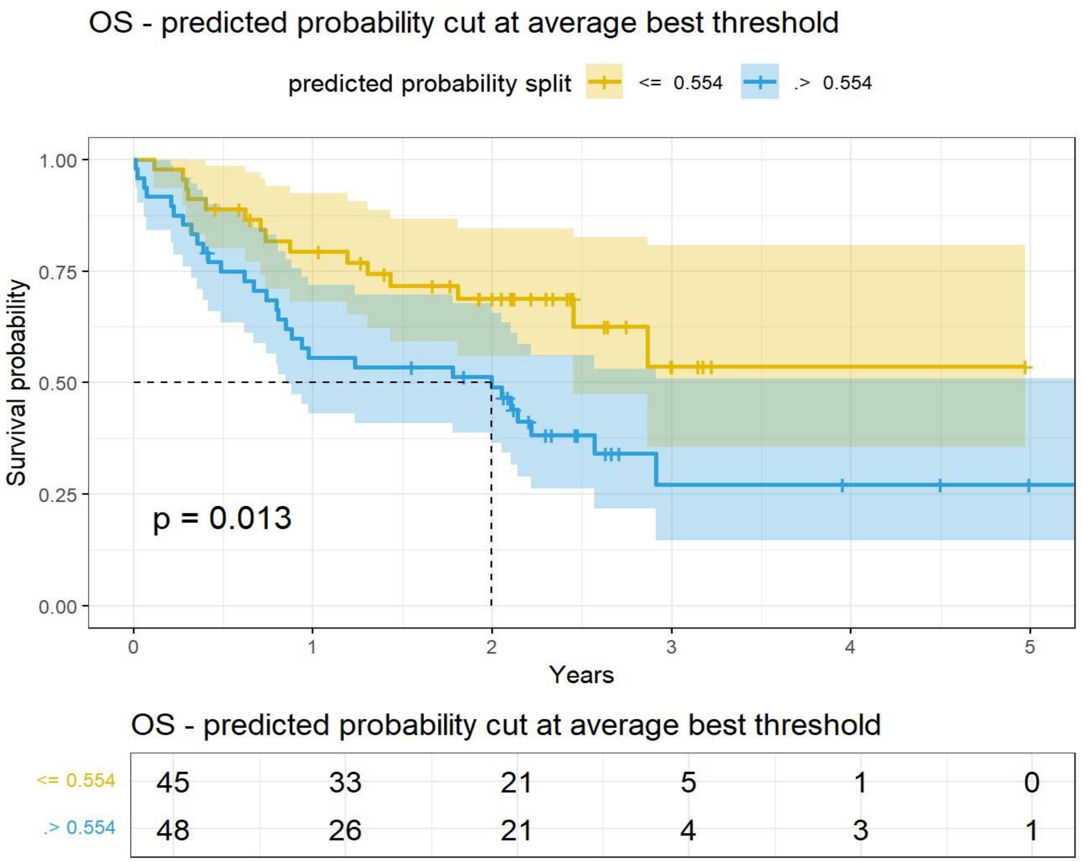

(b)

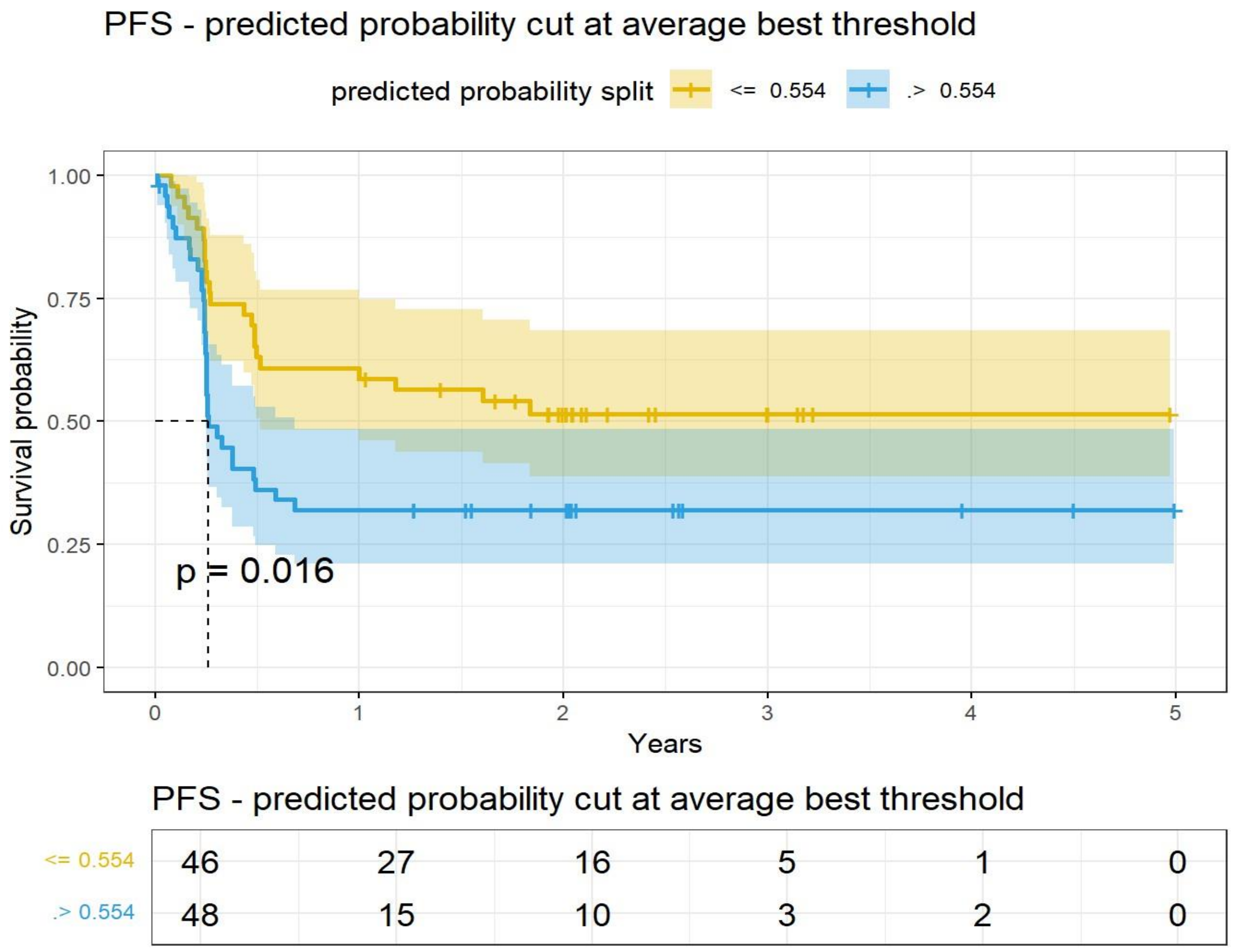

PFS - predicted probability cut at average best threshold
predicted probability split
<= 0.554
.> 0.554
1.00
0.75
0.50
0.25
0.00
Survival probability
p = 0.016
0
1
2
3
4
5
Years
PFS - predicted probability cut at average best threshold
<= 0.554
46
27
16
5
1
0
.> 0.554
48
15
10
3
2
0

(c)

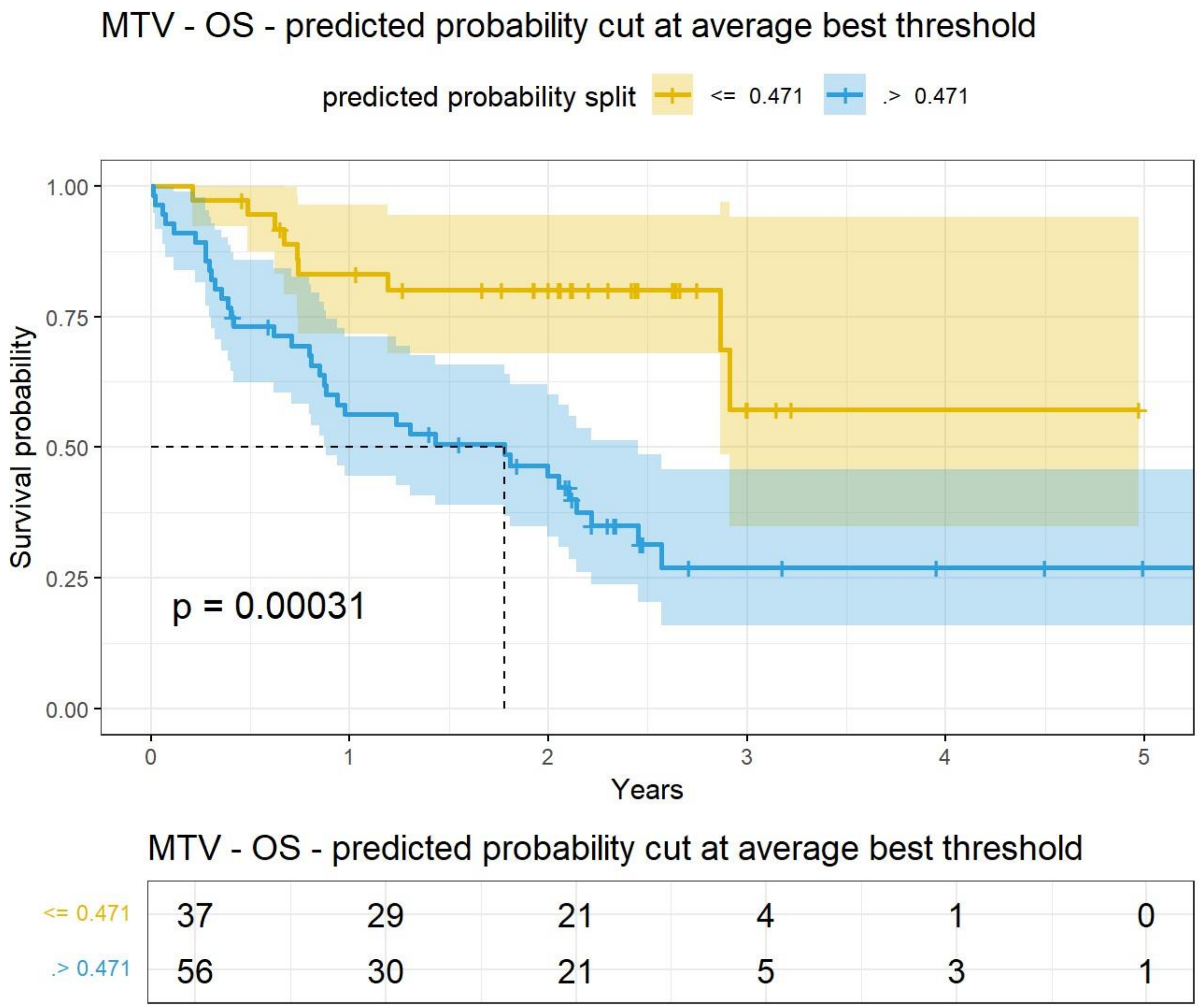
MTV - OS - predicted probability cut at average best threshold
predicted probability split
<= 0.471
.> 0.471
Survival probability
1.00
0.75
0.50
0.25
0.00
p = 0.00031
0
1
2
3
4
5
Years
MTV - OS - predicted probability cut at average best threshold
<= 0.471
37 29 21 4 1 0
.> 0.471
56 30 21 5 3 1

(d)

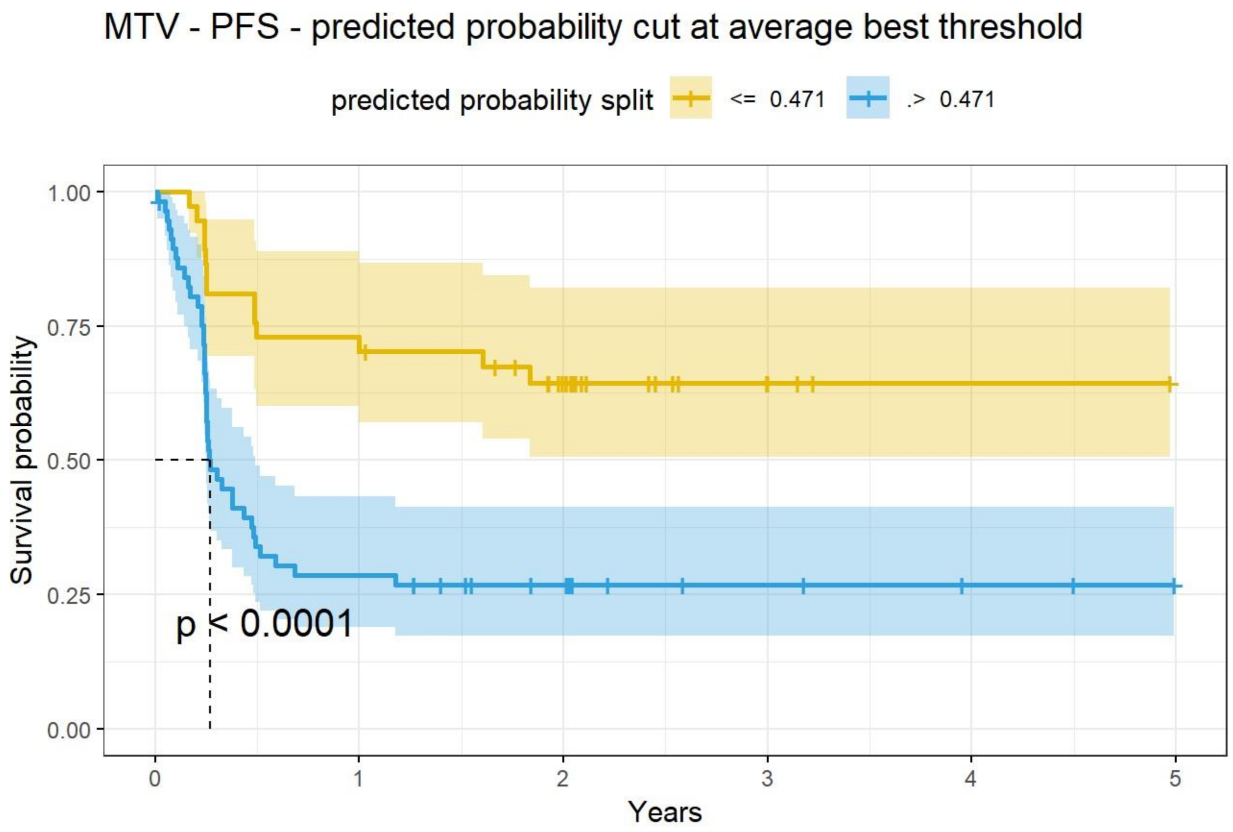

MTV - PFS - predicted probability cut at average best threshold

<= 0.471	37	26	17	4	1	0
.> 0.471	57	16	9	4	2	0

SUPPLEMENTAL REFERENCES:

1. D.-H Xu ASK, J.D. Furst, and D.S. Raicu. Run-length encoding for volumetric texture. presented at: the IASTED Int'l Conf on Visualization, Imaging and Image Processing; 2004;
2. Tang X. Texture information in run-length matrices. *IEEE Transactions on Image Processing*. 1998;7(11):1602-1609.
3. A.S. Kurani D-HX, J.D. Furst, and D.S. Raicu. Co-occurrence matrices for volumetric data. presented at: 7th IASTED Int'l Conf on Computer Graphics and Imaging; 2004;
4. Mokji M.M ABSAR. Gray Level Co-Occurrence Matrix Computation Based On Haar Wavelet. presented at: IEEE Computer Graphics, Imaging and Visualisation (CGIV '07); Aug 2007;
5. V.A. Kovalev FK, H.-J Gertz, and D.Y. von Cramon. Three-dimensional texture analysis of MRI brain datasets. *IEEE Trans on Medical Imaging*. 2001;20(5):424-433.
6. Laws K. *Texture Image Segmentation*. University of South California 1980.
7. Benke K K CDaSDR. A study of the effect of image quality on texture energy measures. *Meas Sci Technol*. 1994;5:400-7.
8. K. Jafari-Khouzani HS-Z, K. Elisevich, and S. Patel. Comparison of 2D and 3D wavelet features for the lateralization. *In Proc of SPIE Medical Imaging 2004: Physiology, Function and Structure from Medical Images*. 2004;5369:593-601.
